# Auxiliary subunits control biophysical properties and response to compound NS5806 of the Kv4 potassium channel complex

**DOI:** 10.1096/fj.201902010RR

**Published:** 2019-11-27

**Authors:** Hongxue Zhang, Hua Zhang, Chanjuan Wang, Yuhong Wang, Ruya Zou, Chenxia Shi, Bingcai Guan, Nikita Gamper, Yanfang Xu

**Affiliations:** ^1^ Department of Pharmacology Hebei Medical University Shijiazhuang China; ^2^ The Key Laboratory of New Drug Pharmacology and Toxicology Ministry of Education Shijiazhuang China; ^3^ The Key Laboratory of Neural and Vascular Biology Ministry of Education Shijiazhuang China; ^4^ Institute of Masteria Medica Chinese Academy of Medical Sciences & Peking Union Medical College Beijing China; ^5^ School of Biomedical Sciences Faculty of Biological Sciences University of Leeds Leeds UK

**Keywords:** cardiomyocyte, dipeptidyl peptidase‐like protein, K^+^ channel‐interacting protein, transient outward K^+^ current

## Abstract

Kv4 pore‐forming subunits co‐assemble with β‐subunits including KChIP2 and DPP6 and the resultant complexes conduct cardiac transient outward K^+^ current (*I*_to_). Compound NS5806 has been shown to potentate *I*_to_ in canine cardiomyocytes; however, its effects on *I*_to_ in other species yet to be determined. We found that NS5806 inhibited native *I*_to_ in a concentration‐dependent manner (0.1~30 μM) in both mouse ventricular cardiomyocytes and human‐induced pluripotent stem cell‐derived cardiomyocytes (hiPSC‐CMs), but potentiated *I*_to_ in the canine cardiomyocytes. In HEK293 cells co‐transfected with cloned Kv4.3 (or Kv4.2) and β‐subunit KChIP2, NS5806 significantly increased the peak current amplitude and slowed the inactivation. In contrast, NS5806 suppressed the current and accelerated inactivation of the channels when cells were co‐transfected with Kv4.3 (or Kv4.2), KChIP2 and another β‐subunit, DPP6‐L (long isoform). Western blot analysis showed that DPP6‐L was dominantly expressed in both mouse ventricular myocardium and hiPSC‐CMs, while it was almost undetectable in canine ventricular myocardium. In addition, low level of DPP6‐S expression was found in canine heart, whereas levels of KChIP2 expression were comparable among all three species. siRNA knockdown of DPP6 antagonized the *I*_to_ inhibition by NS5806 in hiPSC‐CMs. Molecular docking simulation suggested that DPP6‐L may associate with KChIP2 subunits. Mutations of putative KChIP2‐interacting residues of DPP6‐L reversed the inhibitory effect of NS5806 into potentiation of the current. We conclude that a pharmacological modulator can elicit opposite regulatory effects on Kv4 channel complex among different species, depending on the presence of distinct β‐subunits. These findings provide novel insight into the molecular design and regulation of cardiac *I*_to_. Since *I*_to_ is a potential therapeutic target for treatment of multiple cardiovascular diseases, our data will facilitate the development of new therapeutic *I*_to_ modulators.

**Abbreviations**
AP: action potential
APD: action potential duration
DPP: dipeptidyl peptidase‐like protein
ECG: electrocardiogram
HEK: human embryonic kidney
HF: heart failure
hiPSC‐CMs: human‐induced Pluripotent Stem Cell‐derived cardiomyocytes
*I*_k,slow_: the slow component of delayed rectifier K^+^ currents
*I*_to_: transient outward K^+^ current
*I*_to,f_: fast component of *I*_to_
*I*_to__,s_: slow component of *I*_to_
KChIP: K^+^ channel‐interacting protein
QTc: heart rate–corrected QT intervals
siRNA: small interfering RNA

## 1 INTRODUCTION

Kv4 channels are the primary subunits of the rapidly activating and inactivating K^+^ channels, contributing to the cardiac transient outward K^+^ currents (*I*_to_).^1^ Due to its unique kinetic features, Kv4 channels play a central role in controlling cardiac excitation and shaping the cardiac action potentials (AP). The reduction of the *I*_to_ density and the consequent prolongation of the action potential duration is a consistent finding in many pathological conditions such as cardiac hypertrophy and heart failure (HF) and may contribute to the arrhythmias.^2^, ^3^ Hence, the pharmacological *I*_to_ activation may have therapeutic value in HF.

It is generally accepted that there are two *I*_to_ components with distinct recovery kinetics: the fast (*I*_to,f_) and slow (*I*_to,s_).^4^ The Kv4.2 (*KCND2*) and Kv4.3 (*KCND3*) conduct *I*_to,f_, while Kv1.4 (*KCNA4*) forms *I*_to,s_ channels. In human and canine ventricular myocytes, functional *I*_to,f_ channels are likely to be assembled from Kv4.3 homotetramers because Kv4.2 is not expressed.^5^, ^6^ In mice, Kv4.2 is believed to be the principal α‐subunit of *I*_to,f_.^7^ Auxiliary subunits, such as K^+^ channel‐interacting proteins (KChIPs) or dipeptidyl peptidase‐like proteins (DPPs), are known to interact with and modulate the properties of Kv4 channels.^8^, ^9^ Within these, KChIP2 and DPP6 have been proposed as most likely candidates that co‐assemble with Kv4 subunits in the human heart.^9^ KChIP2 is a cytosolic Ca^2+^‐binding auxiliary subunit that interacts with the amino terminus of the Kv4 α‐subunit; it facilitates channel trafficking, slows the Kv4 current inactivation kinetics, accelerates the recovery from inactivation, and shifts the voltage dependence of steady‐state inactivation to more positive potentials.^10^ DPP6 is a single‐membrane‐spanning protein that accelerates current inactivation and recovery kinetics and shifts both activation and inactivation voltage dependencies of Kv4 channels to more negative potentials.^11^ Co‐expression of Kv4.3 with KChIP2 and DPP6 in heterologous systems results in currents with kinetics similar to those of the *I*_to_ in human ventricular myocytes.^9^ Thus, Kv4‐KChIP‐DPP complex could be a valuable target for therapeutic approaches to cardiac diseases.

A small molecule sulfonylurea compound, NS5806, has been found to potentate *I*_to_ in canine cardiac myocytes by slowing the current inactivation. Further studies have shown that current potentiation by NS5806 depends on the presence of KChIPs in the recombinant Kv4 channel complex.^12^, ^13^ NS5806 was shown to reverse the HF‐associated reduction of *I*_to_ density in canine ventricular cardiomyocytes^14^, ^15^ and, thus, NS5806 was put forward as a prototypic *I*_to_ opener and a candidate template for antiarrhythmic drugs.^16^ However, its effects on *I*_to_ in other species yet to be determined. Given its proposed therapeutic value, a better understanding of the molecular mechanisms underlying the modulation of Kv4 channels by NS5806 is urgently required.

*I*_to_ is the major component of the cardiac AP repolarization in rodents and the mice have become an important model for the molecular studies of *I*_to_. In addition, human‐induced pluripotent stem cell‐derived cardiomyocytes (hiPSC‐CMs) have recently become another important system for modeling human cardiac disease and drug screening.^17^ In this study, we examined the effect of NS5806 on *I*_to_ in native mouse ventricular cardiomyocytes and in the hiPSC‐CMs. Unexpectedly, we discovered that in contrast to its effect in canine cardiomyocytes, NS5806 decreased the amplitude of native *I*_to_ in both cell types with a significant acceleration of the current inactivation. We used primary cardiac cells from different species, hiPSC‐CMs, expression system, and modeling to probe the intricate molecular mechanisms underlying the species‐specific differences in pharmacological responses of *I*_to_ to NS5806.

## 2 MATERIALS AND METHODS

### 2.1 Animals

Male C57BL/6 mice (weight 20‐25 g, 7‐ to 8‐week old) and male Beagle dogs (weight 6‐8 Kg, 1‐ to 2‐year old) were purchased from the Vital River Laboratory Animal Technology Company (Beijing, China). Animal care standards and all experimental procedures were approved by the Animal Care and Ethical Committee of Hebei Medical University (Shijiazhuang, China). Mice were housed under the specific pathogen‐free (SPF) conditions with five mice per cage and dogs were raised in a conventional animal room. All animals were kept under controlled environmental conditions (12 hours light/12 hours dark cycle, room temperature 21‐23°C and humidity 50%‐60%) with free access to standard laboratory food pellets and water.

### 2.2 Ventricular cardiomyocytes isolation

Single ventricular myocytes were enzymatically isolated from five mouse and three canine hearts as described previously.^18^, ^19^ Briefly, animals were injected with heparin (1.0 U·kg^−1^) and anaesthetized with sodium pentobarbital (35 mg·kg^−1^). Hearts were rapidly removed, and a wedge from canine left ventricular free wall supplied by a descending branch of the circumflex artery was excised. Intact mouse heart or wedge preparation from canine heart were mounted on a Langendorff apparatus (Radnoti Inc, Monrovia, CA, USA), and retrogradely perfused through the aorta with Ca^2+^‐free Tyrode's solution (mM): NaCl 140, KCl 5.4, MgCl_2_ 1, HEPES 10, and glucose 10 (pH 7.4 with NaOH). After 5 minutes of perfusion, the same solution supplemented with Type II collagenase (Worthington Biochemical Corporation, Lakewood, NJ, USA; 0.4 mg·mL^−1^) was applied for 10‐15 minutes. Once ventricular tissue was softened, hearts were removed from the apparatus. The left apex ventricles of mouse heart or canine epicardium ventricles were sliced into small pieces and incubated in KB solution (in mM): KOH 80, KCl 40, KH_2_PO_4_ 25, MgSO_4_ 3, Glutamic acid 50, Taurine 20, HEPES 10, EGTA 1, and Glucose 10 (pH 7.4 adjusted with KOH). Cells were then harvested and were used for patch‐clamp recordings within 4‐6 hours after isolation.

### 2.3 In vitro electrocardiographic (ECG) recordings

The Langendorff hearts from five mice were prepared with the above method and were retrogradely perfused (2‐2.5 ml·min^−1^; 37 ± 0.5°C) through the aorta with oxygenated Tyrode's solution (in mM): NaCl 140, KCl 5.4, MgCl_2_ 1, CaCl_2_ 2, HEPES 10, and glucose 10 (pH 7.4 adjusted with NaOH) using peristaltic pump. The hearts were put in the thermostatic chamber and allowed to equilibrate for a minimum of 30 minutes to ensure the stable ECG recordings before drug testing. The in vitro equivalent lead II ECG waveforms were recorded by the Biopac 150 System (Biopac Systems, USA) at 5 kHz. The QT interval was defined as the time between the first deviation from the isoelectric line during the PQ interval until the end of the T wave, and was averaged from 30 consecutive beats between 5 and 10 minutes after the presence of test compound. The heart rate was defined by RR intervals. The heart rate–corrected QT intervals (QTc) were calculated according to a parabolic equation QT = (333/RR)^0.601^.^20^

### 2.4 cDNA constructs and mutagenesis

cDNAs coding for human Kv4.3 (NM_172198), Kv4.2 (NM_012281), KChIP2 (NM_173192), DPP6‐L (long isoform; NM_130797), and DPP6‐S (short isoform; NM_001936) were cloned into pcDNA3.1 by Youbio (Changsha, China). Alanine substitution at positions R7, P33, D36, G38, L44 in DPP6‐L was performed by Youbio. Validity of all cDNA constructs was confirmed by sequencing.

### 2.5 Cell culture, transfection, and siRNA treatment

Human embryonic kidney (HEK) 293 cells were cultured in Dulbecco’s modified Eagle’s medium (DMEM, Gibco) supplemented with 10% fetal calf serum (Gibco) with 5% CO_2_ at 37°C. The HEK293 cells were transiently co‐transfected with Kv4.3 (or Kv4.2), KChIP2, and DPP6 plasmid using Lipofectamine 2000 (Invitrogen) according to the manufacturer’s instructions. The green fluorescent protein (GFP) was used as a reporter. The human‐induced pluripotent stem cell‐derived cardiomyocytes (hiPSC‐CMs, 50‐60 days of maturity; Cellapybio, Beijing, China) were maintained in serum‐free medium (Cellapybio) on 24‐well dishes coated with Matrigel Matrix (BD‐Biocoat, Corning, USA) with 5% CO_2_ at 37°C. The small interfering RNA (siRNA) duplex against the human DPP6 gene (NM_130797, 5′‐GGTCCATCATCGGCTCTTT‐3′) was designed and constructed by Ribobio (Guangdong, China). hiPSC‐CMs were transfected with siRNA using Lipofectamine RNAiMax (Invitrogen) according to the manufacturer’s instruction at a final siRNA concentration of 100 nM. A non‐matching siRNA was used as a negative control. Cells were used for experiments 48 hours after transfection.

### 2.6 Patch‐clamp recordings

The conventional whole‐cell patch‐clamp recordings were performed at room temperature (22‐25°C) using an EPC‐10 amplifier in combination with Patchmaster software (V2x73.2, HEKA, Lambrecht, Germany) and data analysis was performed using Origin 8.6 software (Wavemetrics, Microcal, USA). The access resistance was typically within 5 MΩ. Whole‐cell membrane capacitances were cancelled and series resistance compensated by 80%. For native *I*_to_ recording, the external solution contained (in mM) N‐methyl‐d‐glucamine (NMG) 130, KCl 5, MgCl_2_ 1, CaCl_2_ 1, HEPES 10, and glucose 10 (pH 7.4 with HCl) and the pipette solution contained (in mM) KCl 140, Mg‐ATP 4, MgCl_2_ 1, EGTA 10, and HEPES 10 (pH 7.4 with KOH). The CdCl_2_ (300 nM) was added in the bath solution to block voltage‐gated Ca^2+^ channels. For recording from HEK293 cells, the extracellular solution contained (in mM) NaCl 140, KCl 5, CaCl_2_ 1.8, MgCl_2_ 1.2, HEPES 10, and Glucose 10 (pH 7.4 with NaOH) and the patch pipette solution contained (in mM) KCl 140, Mg‐ATP 4, MgCl_2_ 1, EGTA 10, and HEPES 10 (pH 7.4 with KOH). To isolate distinct K^+^ current components, a rapidly activating and inactivating current, *I*_to,f_; a rapidly activating but slowly inactivating current, *I*_k,slow_; and a slowly activating non‐inactivating current, *I*_ss,_ the decay phases of the currents evoked during long (4.5 seconds) depolarizing voltage steps to various test potentials were fitted by the sum of two exponents. The following equation was used: *y*(*t*) = *A*_1_·exp(−*t*/*τ*1) + *A*_2_·exp(−*t*/*τ*_2_) + *A*_ss_., where *t* is time, *τ*_1_ and *τ*_2_ are, respectively, the time constants of decay of *I*_to,f_ and *I*_k,slow_; *A*_1_ and *A*_2_ are, respectively, the amplitudes of *I*_to,f_ and *I*_k,slow_, and *A*_ss_ is the amplitude of *I*_ss_.^21^

### 2.7 Western blot and immunoprecipitation assays

Immunoblots were performed as previously described.^22^ Proteins were prepared from the left ventricular free wall of five mouse hearts, epicardial tissue of three dog hearts, and three batches of hiPSC‐CMs (50‐60 days of maturity). Protein concentration was measured by BCA protein assay (Pierce, USA). Denatured samples (60 μg/lane) were separated on precast 10% SDS‐PAGE gels and transferred to PVDF membranes. Quantification of the signals was performed by Odyssey Infrared Imaging System (LICOR 9120, Li‐COR, Lincoln, NE, USA). Densitometry was used for quantification; the protein band pixel intensities were normalized to the GAPDH band in each sample. The values were then averaged from all the different sets of experiments. The following primary antibodies were used: anti‐DPP6 (a polyclonal antibody raised in rabbit against a purified peptide corresponding to amino acid residues 400‐550 of human DPP6, Abcam, UK); anti‐Kv1.4 (a polyclonal antibody raised in rabbit against a purified peptide corresponding to amino acid residues 589‐655 of rat Kv1.4 located in intracellular C‐terminus); anti‐Kv4.3 (a polyclonal antibody raised in rabbit against a purified peptide corresponding to amino acid residues 451‐468 of human Kv4.3 located in intracellular C‐terminus); anti‐KChIP2 (a polyclonal antibody raised in rabbit against a purified peptide corresponding to amino acid residues 2‐15 of human KChIP2 located in intracellular N‐terminus) (Alomone Labs, Israel); and anti‐GAPDH (Proteintech, Wuhan, China).

### 2.8 RT‐PCR

Total RNA from hiPSC‐CMs was extracted using Trizol Reagent (Takara, Japan). cDNA was synthesized using the PrimeScript RT Reagent Kit (Takara, Japan). Real‐time PCR was performed using ABI Prism 7300 Real‐Time PCR System (ABI, Wilmington, USA). The following primers (synthesized by Sangon Biotech, Shanghai, China) were used: GAPDH forward: 5′‐AAGACCTTGGGCTGGGACT‐3′; GAPDH reverse: 5′‐CCAAATCCGTTGACTCCGAC3′. DPP6 forward: 5′‐AAAGAGAGAAAGCACAGCCAGAG‐3′; DPP6 reverse: 5′‐CTTCTGGTGGGTCCAGACTTT‐3′. The amplification curves to provide Ct values were normalized to the reference gene GAPDH; the changes in expression were calculated using the 2^−ΔΔCT^ method.

### 2.9 Computational prediction of interaction between KChIP2 and DPP6

Computer simulations and analyses were performed by Beijing Abace Biotechnology Co., Ltd. BLAST and HHBlist were used with default parameters to identify evolutionary related structures matching the KChIP2 sequence (UniProtKB accession number Q9NS61). Kv channel‐interacting protein 4 (KChIP4a; PDB ID: 3DD4) was identified as the highest ranked homolog. A known structure of KChIP4a,^23^ which had 80% sequence identity with KChIP2, was thus used as a template to generate a homology model of KChIP2. No appropriate templates were identified for DPP6‐L in the Protein Data Bank. Therefore, *ab initio* protein prediction approaches were employed for structure modeling of DPP6‐Lin. The docking experiment was performed using the program GRAMM. A global search on the surface of KChIP2 was done to identify potential binding sites for DPP6‐Lin. All the predicted binding positions were ranked based on their docking scores. As a result, DPP6‐Lin was successfully docked into KChIP2 and a total of 10 models were generated. The interactions between DPP6‐L and KChIP2 were analyzed using the PDBePISA web server.

### 2.10 Chemicals

NS5806 (Tocris, UK) was prepared as a 20 mM stock solution in DMSO and stored in −20°C. The highest final concentration of DMSO in external solution was ≤0.1%, a concentration that had no effect on the current recording. Phrixotoxin‐2 (Abcam, UK) was prepared as a 1 mM stock solution in water and stored at −20°C. Other common chemicals were from Sigma.

### 2.11 Data and statistical analysis

All data are presented as Mean ± SEM. Data analysis and statistics were carried out using Origin 8.6 software (OriginLab Corporation, USA). Group comparisons were performed with paired Student’s *t* tests (for comparisons before and after drug treatment) and unpaired Student’s *t* tests (for two‐group comparisons) and ANOVA with Dunnett’s post hoc tests (for multiple‐group comparisons). The “n” and “N” were used to represent the number of cells and animals/samples, respectively. Post hoc tests were only performed when *F* achieved *P* < .05 and there was no significant variance in the homogeneity. The differences were considered significant at *P* < .05.

## 3 RESULTS

### 3.1 Effect of NS5806 on outward potassium current in mouse left ventricular myocytes

**Figure 1:**
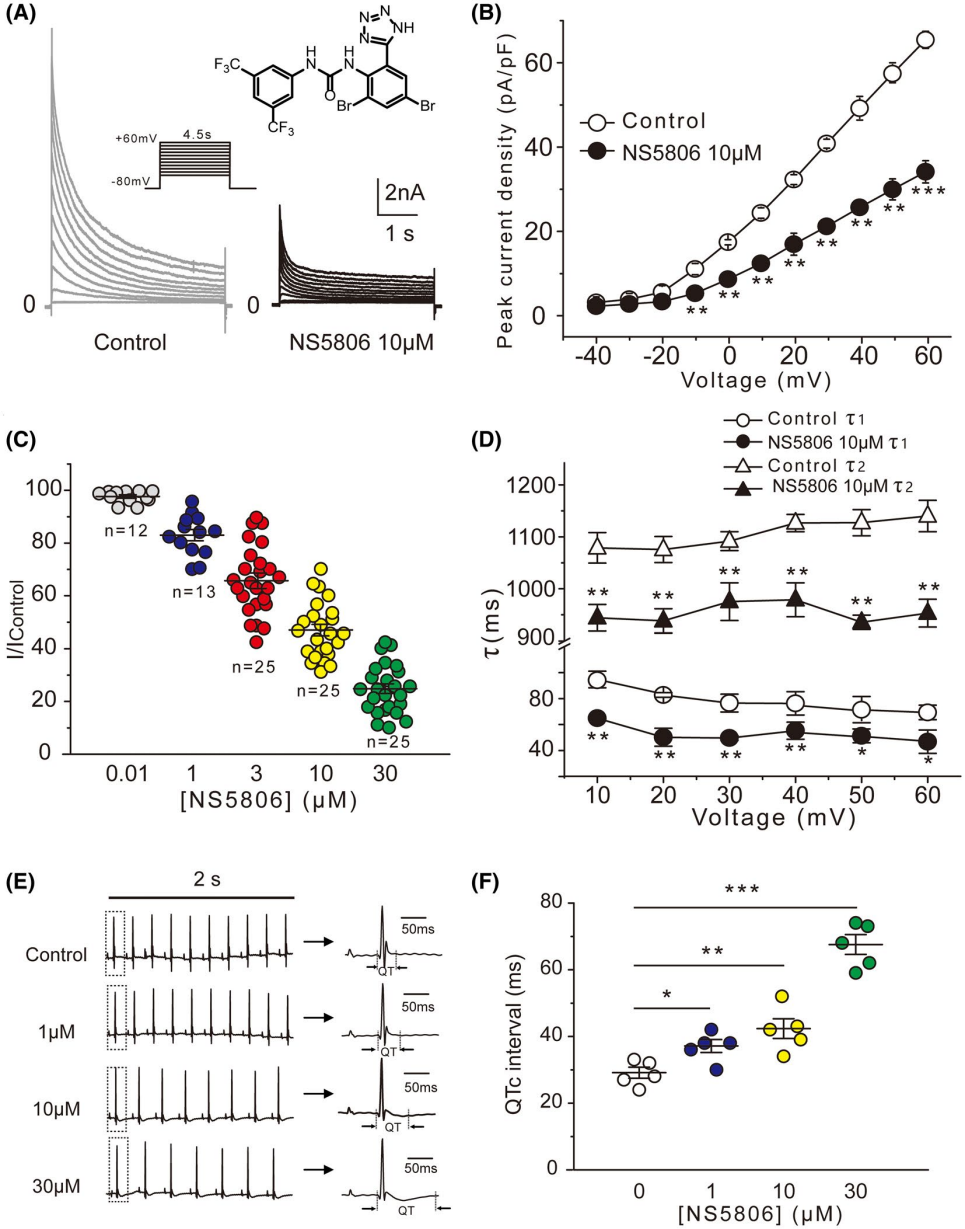
Effects of NS5806 on the outward potassium currents in mouse left ventricular myocytes. A, Representative currents recorded in the absence (left) and presence of 10 μM NS5806 (right) using voltage protocol depicted in the inset. The molecular structure of NS5805 is shown in the upper inset. B, I–V relationships of the peak outward current (expressed as current density) before and after the application of 10 μM NS5806 (n = 25, N = 5, **P < .01, ***P < .001 vs Control). C, Concentration dependence of the effect of NS5806 on outward potassium currents; the IC_50_ = 6.6 μM. The number under each dataset represents the number of cardiomyocytes (from five hearts) tested at each concentration. D, Effect of NS5806 on the voltage dependence of inactivation. The inactivation phases of outward potassium current at +60 mV were fitted by double‐exponential function to provide the fast (*τ*_1_) and slow (*τ*_2_) time constants (n = 25, N = 5, *P < .05, **P < .01 vs Control). E, Representative ECG waveforms from Lengendorff perfused mouse hearts at different concentrations of NS5806 (left). The arrows indicate the representative ECG waveforms enlarged from the boxed area of each corresponding trace. F, Histogram of Mean (± SEM) QTc intervals from recordings as in E (N = 5, *P < .05, **P < .01, ***P < .001 vs Control)

We first tested the effect of NS5806 (Figure 1A, inset) on the outward potassium currents in mouse ventricular myocytes. The currents were recorded using a series of long (4.5 seconds) depolarizing voltage steps from −40 to +60 mV from a holding potential of −80 mV. NS5806 significantly suppressed the outward potassium currents; the peak current density was significantly decreased at all potentials positive to −10 mV (Figure 1A,B). The inhibition was concentration‐dependent (Figure 1C). Current inactivation was well fitted with a double‐exponential equation providing rapid and slow inactivation time constants, *τ*_1_ and *τ*_2_, respectively.^21^ NS5806 induced significant acceleration of the current inactivation; at +60 mV *τ*_1_ changed from the control value of 53.8 ± 5.5 to 41.8 ± 3.0 ms and *τ*_2_ from 1279.8 ± 60.3 to 906.3 ± 53.7 ms (Figure 1D). The effect of NS5806 on cardiac electrophysiological activity was further tested in isolated perfused mouse hearts. Representative in vitro ECG waves in the presence of NS5806 at different concentrations are shown in Figure 1E. Analysis of ECG recordings revealed that NS5806 significantly prolonged the heart rate–corrected QT interval (QTc) in a concentration‐dependent manner (Figure 1F). These results indicated that NS5806 suppressed repolarizing currents and thus prolonged QT interval in mouse heart.

**Figure 2:**
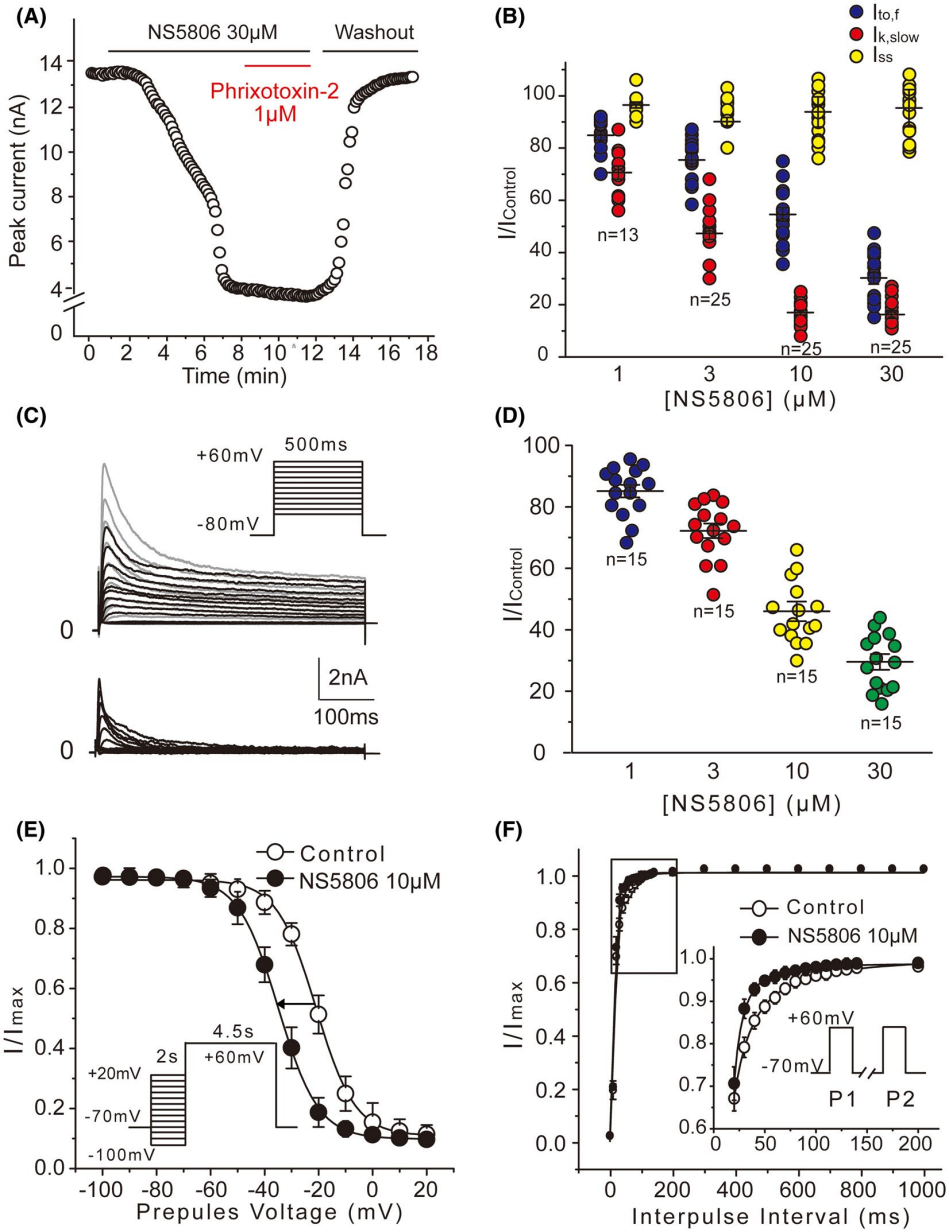
Effects of NS5806 on different K^+^ current components in mouse left ventricular myocytes. A, Time course of response of outward K^+^ current to Phrixotoxin‐2 (1 μM) in the presence of NS5806 (30 μM). B, Concentration‐dependent effect of NS5806 on the three components of K^+^ current: *I*_to,f_, *I*_k,slow_, and *I*_ss_ (see Methods). C, Representative outward potassium currents were recorded in the absence (grey) and presence (black) of 10 μM NS5806. Currents were evoked by 500 ms square voltage steps (inset); corresponding NS5806‐sensitive currents (bottom) were acquired through digital subtraction. D, Concentration‐dependent effect of NS5806 on *I*_to,f_ recorded under the pulse protocol shown in C; n = 15, N = 5. E, Steady‐state inactivation of *I*_to,f_ in the absence and presence of NS5806 (10 μM) using the conditioning pre‐pulse protocol (inset); the data were fitted to Boltzmann equation (n = 25). F, *I*_to,f_ recovery from steady‐state inactivation recorded using a double‐pulse protocol with variable inter‐pulse intervals (inset) in the absence and presence of NS5806 (n = 25, N = 5)

The outward potassium currents in adult mouse myocytes from free left ventricular wall has three distinct components: a rapidly activating and inactivating, *I*_to,f_; a rapidly activating but slowly inactivating current, *I*_k,slow_; and a slowly activating non‐inactivating current, *I*_ss_.^21^ To isolate these components, we used two‐exponent fit of current inactivation (see above and Methods) and selective pharmacology. Phrixotoxin‐2, a specific Kv4 channel blocker,^24^ selectively blocked the *I*_to,f_ in mouse ventricular cardiomyocytes in a concentration‐dependent manner; at 1 μM Phrixotoxin‐2 almost completely suppressed *I*_to,f_ (Figure S1). Interestingly, after the application of 30 μM NS5806, 1 μM Phrixotoxin‐2 failed to inhibit remaining K^+^ current, suggesting that NS5806 suppressed the majority of Kv4‐dependent fraction of *I*_to,f_ (Figure 2A). Further analysis revealed that NS5806 decreased the current amplitude of both *I*_to,f_ and *I*_k,slow_ in a concentration‐dependent manner, but it did not affect *I*_ss_ (Figure 2B). The IC_50_ values for the inhibition of *I*_to,f_ and *I*_k,slow_ were 12.5 ± 0.2 and 2.7 ± 0.7 μM, respectively. We then used shorter (500 ms) depolarizing pulses (Figure 2C), as under such conditions the peak current amplitude (with the sustained component subtracted) was shown to represent “pure” *I*_to,f_.^25^ Figure 2C shows representative current traces before and after the application of 10 μM NS5806 (top panel), as well as the NS5806‐sensitive current fraction (bottom panel). The inactivation phase of the NS5806‐sensitive current fraction was well fitted by a single exponential equation, which gave time constants within the same range as *τ*_1_ provided by double‐exponential fitting under long depolarization pulse (data not shown). Under these conditions, NS5806 inhibited *I*_to,f_, with the IC_50_ of 10.9 ± 0.5 μM (Figure 2D; cf. to IC_50_ of 12.5 ± 0.2 μM obtained from long depolarization pulse recordings). These data confirmed that NS5806 suppressed *I*_to,f_ in mouse cardiomyocytes. *I*_k,slow_ was also inhibited but *I*_ss_ was not affected.

The effect of NS5806 on the steady‐state inactivation of *I*_to,f_ was then examined under the voltage protocol shown in the inset in Figure 2E. The inactivation phases of the currents evoked at +60 mV after each pre‐pulse potential were fitted by double‐exponential equation to quantify the effect on *I*_to,f_. NS5806 induced significant leftward shift of the voltage dependence of steady‐state inactivation with the half‐maximal voltage (*V*_1/2_) shifted from −20.9 ± 0.7 mV under control conditions to −34.5 ± 0.8 mV in the presence of the drug. The recovery from inactivation of *I*_to,f_ was evaluated by a paired‐pulse voltage clamp protocol (Figure 2F, inset). The first inactivating pulse to +60 mV (4.5 seconds) was followed by variable repolarization periods (0‐2000 ms), with subsequent second pulse to +60 mV. The amplitudes of *I*_to,f_ were obtained by two‐exponential fitting and then normalized to their respective maximal current amplitudes. The recovery from inactivation was well fitted by single‐exponential equation (Figure 2F); NS5806 significantly accelerated the recovery with the time constant changing from 24.5 ± 1.3 to 11.6 ± 1.0 ms.

### 3.2 Effect of NS5806 on *I*_to_ in hiPSC‐CMs

**Figure 3:**
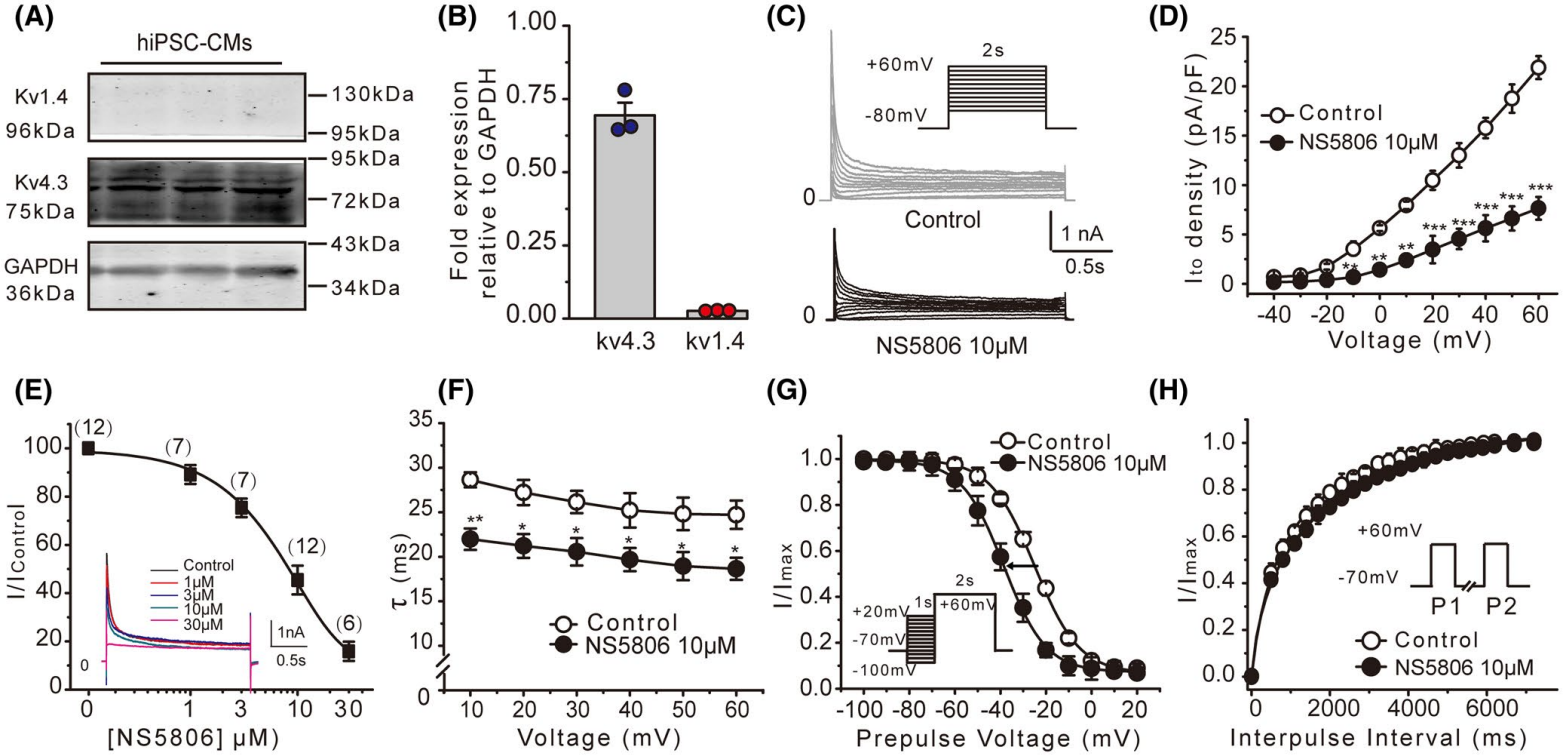
Effects of NS5806 on *I*_to_ in human‐induced pluripotent stem cell‐derived cardiomyocytes (hiPSC‐CMs). A, Representative western blot analysis of Kv4.3 and Kv1.4 in hiPSC‐CMs. B, Summary data for Kv4.3 and Kv1.4 western blot densitometry normalized to GAPDH (N = 3). C, Representative recordings of *I*_to_ before and after NS5806 (10 μM) using protocol shown in the inset. D, I–V relationships of *I*_to_ currents recorded as in C before and after NS5806 (n = 12, *P < .05, **P < .01, ***P < .001 vs control). E, Concentration‐dependent inhibition of *I*_to_ by NS5806. Data were fitted with a Hill equation; IC_50_ = 8.3 μM. The number above each data point represents the number of cells at each concentration. Representative *I*_to_ traces for each concentration are shown in the inset. F, Time constants (*τ*) of the current inactivation in the absence and presence of NS5806. Data were obtained by fitting current traces to a single exponential equation (n = 12, *P < .05, **P < .01 vs control). G, Steady‐state inactivation of *I*_to_ in the absence and presence of NS5806 recorded using the protocol shown in the inset; data were fitted to Boltzmann equations (n = 12). H, Summary data for *I*_to_ recovery from inactivation in the absence and presence of NS5806 (n = 12)

To identify whether the inhibitory effect of NS5806 on Kv4 channels was just limited to rodents, we investigated the effect of NS5806 on *I*_to_ in hiPSC‐CMs. The molecular correlates of *I*_to_ channel were first assessed using western blot (Figure 3A,B), which revealed that Kv4.3 was the dominant subunit and Kv1.4 was almost undetectable. However, we found that there were comparable expression levels of Kv1.4 in mouse and canine heart (Figure S2). Similar to murine cardiomyocytes, NS5806 induced prominent inhibitory effect in hiPSC‐CMs. Representative *I*_to_ traces before and after NS5806 are shown in Figure 3C. The amplitude of *I*_to_ (peak minus steady‐state current) was significantly inhibited by 10 μM NS5806, with a decrease of 64.6 ± 5.9% from 21.9 ± 1.5 pA/pF (control) to 7.6 ± 1.2 pA/pF in the presence of the drug (measured at +60 mV; Figure 3C). The effect was concentration‐dependent with an IC_50_ of 8.3 ± 1.9 μM (Figure 3E). The current inactivation phase was best fit with a single exponential equation; the *I*_to_ inactivation kinetics was significantly accelerated over a voltage range from +10 to +60 mV, as reflected by the decrease of *τ* values from 45.8 ± 3.5 to 32.6 ± 2.0 ms at +60 mV (Figure 3F). The steady‐state inactivation curves were also analyzed. Consistently, NS5806 shifted the *V*_1/2_ toward negative potentials (from −24.9 ± 0.4 to −38.5 ± 0.3 mV; Figure 3G). However, NS5806 did not significantly affect the recovery from inactivation of *I*_to_ (Figure 3H). These results indicated that NS5806 significantly suppressed *I*_to_ conducted by Kv4 family channels through accelerating current inactivation in hiPSC‐CMs.

Given the striking difference between the effects of NS5806 in canine cardiomyocytes^14^, ^15^ and murine and human cardiomyocytes (present study), we tested the effect of NS5806 on the *I*_to_ in canine cardiomyocytes to confirm its potentiating effect under our experimental conditions. Indeed, the *I*_to_ amplitude was enhanced and the current inactivation was delayed in the presence of 10 μM NS5806 (Figure S3), which concurs with the previous reports.

### 3.3 Effect of NS5806 on the cloned Kv4 channels in heterologous expression system

**Figure 4:**
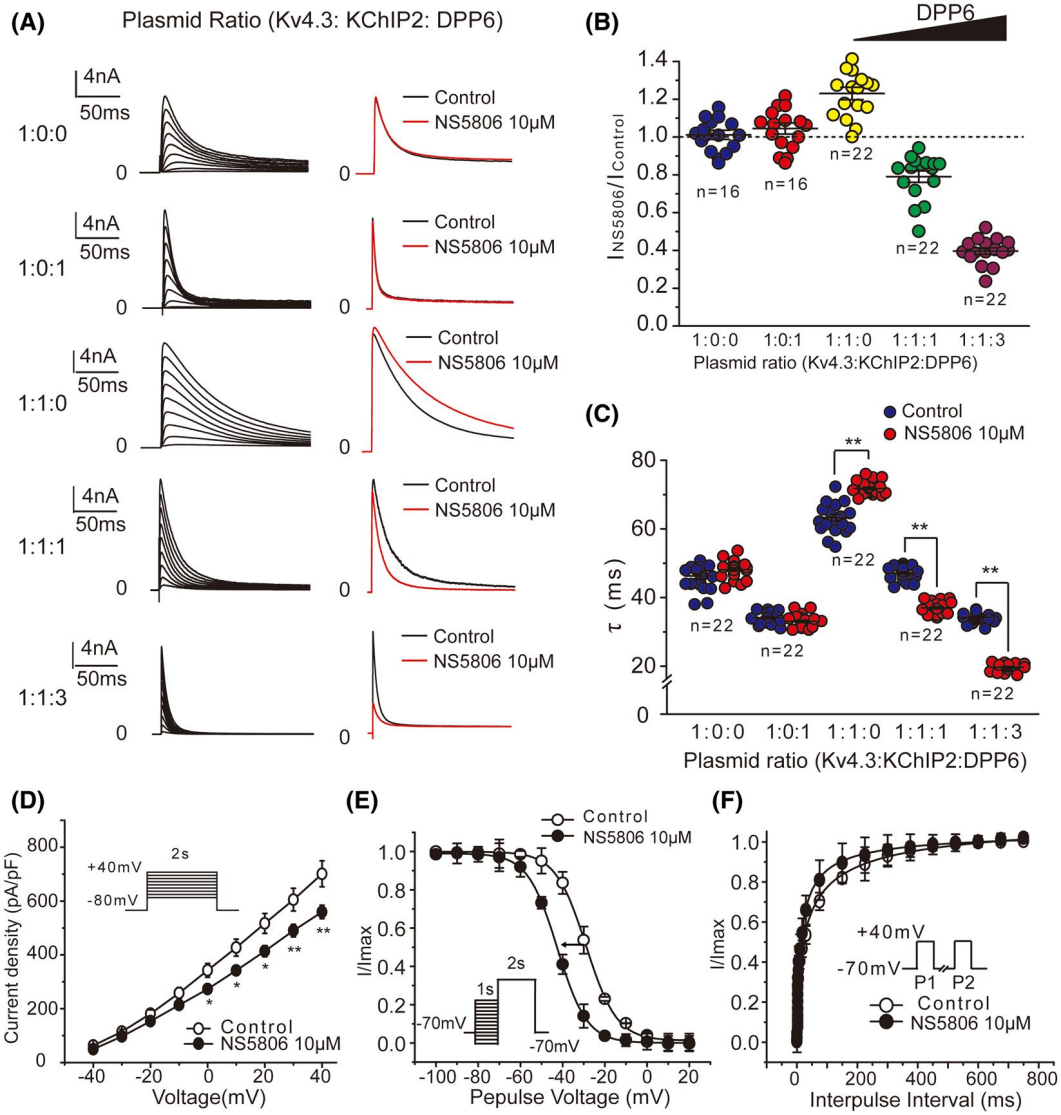
Effects of NS5806 on cloned Kv4.3/KChIP2/DPP6‐L channels in HEK293 cells. A, Representative current traces elicited by the depolarizing voltage steps from −40 to +40 mV for 2 seconds from a holding potential of −80 mV at different transfection ratios of Kv4.3: KChIP2: DPP6‐L (Left). The superimposed current traces at +40 mV in the absence and presence of NS5806 are shown on the right. B, Effect of 10 μM NS5806 on the peak current of the Kv4.3/KChIP2/DPP6‐L currents produced by different subunit transfection ratios, measured at +40 mV. C, The time constant of inactivation (*τ*) of Kv4.3/KChIP2/DPP6‐L currents produced by different subunit transfection ratios (n = 22). D, I–V relationships of Kv4.3/KChIP2/DPP6‐L peak current density at plasmid ratio 1:1:1 before and after 10 μM NS5806 (n = 18, *P < .05, **P < .01 vs control). E, Steady‐state inactivation curves for Kv4.3/KChIP2/DPP6‐L channel complex at plasmid ratio 1:1:1 before and after 10 μM NS5806 (n = 18). F, Recovery from inactivation curves for Kv4.3/KChIP2/DPP6‐L channel complex at plasmid ratio1:1:1 before and after 10 μM NS5806 (n = 18)

To explore the molecular mechanism(s) underlying strikingly different effects of NS5806 on the cardiac *I*_to_ in different species, we next tested the effect of the compound on cloned Kv4.3 channel complex by transiently co‐expressing the pore‐forming α‐subunit with auxiliary subunits KChIP2 and DPP6 at different stoichiometry in HEK293 cells. At least two DPP6 isoforms have been reported: DPP6‐L (long) and DPP6‐S (short). These isoforms differ in the sequence and length of the cytoplasmic N‐terminal domain but share an identical transmembrane domain and a long C‐terminal extracellular domain.^26^ Two isoforms have qualitatively similar regulatory effects on Kv4.2 channels.^27^ DPP6‐S has been used to test the effect of NS5806 on recombinant channel complexes in previous reports, which have demonstrated that NS5806 enhances the current amplitude and concomitantly slows the inactivation kinetics of Kv4.3/KChIP2/DPP6‐S^12^ or Kv4.2/DPP6‐S associated with either KChIP2, KChIP3, or KChIP4 channel complexes.^28^ A similar response was observed in this study when the DPP6‐S was co‐expressed with Kv4.3 and KChIP2 (Figure S4). While consistent with previous observations, this recombinant Kv4.3/KChIP2/DPP6‐S complex did not recapitulate the response to NS5806 of native *I*_to_ from mouse cardiomyocytes or hiPSC‐CMs. To test whether different isoforms of DPP6 confer differential responses to NS5806, the DPP6‐L was co‐transfected with Kv4.3 and KChIP2 in HEK293 cells. As shown in Figure 4A (left panels), the different instantaneous inactivation kinetics were observed when pore‐forming subunit Kv4.3 was co‐assembled with auxiliary subunits KChIP2 or DPP6‐L at different expression ratios. Evidently, DPP6‐L accelerated the current inactivation while KChIP2 slowed inactivation. These findings were consistent with the previous reports.^10^, ^11^ Representative current traces at +40 mV in the absence and presence of NS5806 (10 μM) are shown in Figure 4A (right panels). NS5806 had no effect on the current amplitude of *I*_Kv4.3_ and *I*_Kv4.3/DPP6‐L_, but it enhanced the peak current of *I*_Kv4.3/KChIP2_ at test voltages positive to +10 mV (Figure 4A; Table S1). Intriguingly, NS5806 significantly decreased the peak current of *I*_Kv4.3/KChIP2/DPP6‐L_, and the effect was more prominent when the expression of DPP6‐L was increased by changing Kv4.3/KChIP2/DPP6‐L transfection plasmid ratio from 1:1:1 to 1:1:3 (Figure 4B; Table S2). These results clearly demonstrated that NS5806 significantly enhanced the current amplitude of Kv4.3/KChIP2 channels while inhibiting the current of Kv4.3/KChIP2/DPP6‐L channels. Interestingly, Kv4.3/DPP6‐L channels were unaffected by NS5806, suggesting that an interplay between KChIP2 and DPP6‐L is necessary to confer the inhibition.

Similarly, complex effects of NS5806 on the inactivation kinetics of the cloned channels with the different subunit stoichiometry were observed. As shown in Figure 4C, NS5806 did not affect inactivation time constants of *I*_Kv4.3_ or *I*_Kv4.3/DPP6‐L_, but it significantly delayed the inactivation kinetics of *I*_Kv4.3/KChIP2_. In contrast, NS5806 accelerated the current inactivation in the presence of DPP6‐L subunits and the effect was stronger at the 1:1:3 plasmid ratio of Kv4.3/KChIP2/DPP6‐L, as compared to that at 1:1:1 ratio. NS5806 produced a very similar pattern of effects on Kv4.2/KChIP2/DPP6‐L channels (Figure S5 and Table S3). Taken together, our data suggest that DPP6‐L/KChIP2 levels control the modality of Kv4 channel response to NS5806.

To further test whether the effect of NS5806 in the heterologous system can be closely matched to that in the native cardiomyocytes, we further analyzed its actions on the cloned Kv4.3/KChIP2/DPP6‐L channels at 1:1:1 transfection ratio. These experiments revealed that NS5806 significantly inhibited the currents elicited by voltages positive to −10 mV (Figure 4D). The steady‐state inactivation was left‐shifted with a change of *V*_1/2_ from −28.8 ± 0.4 to −42.5 ± 0.2 mV (Figure 4E), and the recovery from inactivation was accelerated from 30.9 ± 5.1 to 14.4 ± 2.7 ms in the presence of NS5806 (Figure 4F).

### 3.4 The role of DPP6 in the inhibitory action of NS5806 on native *I*_to_

**Figure 5:**
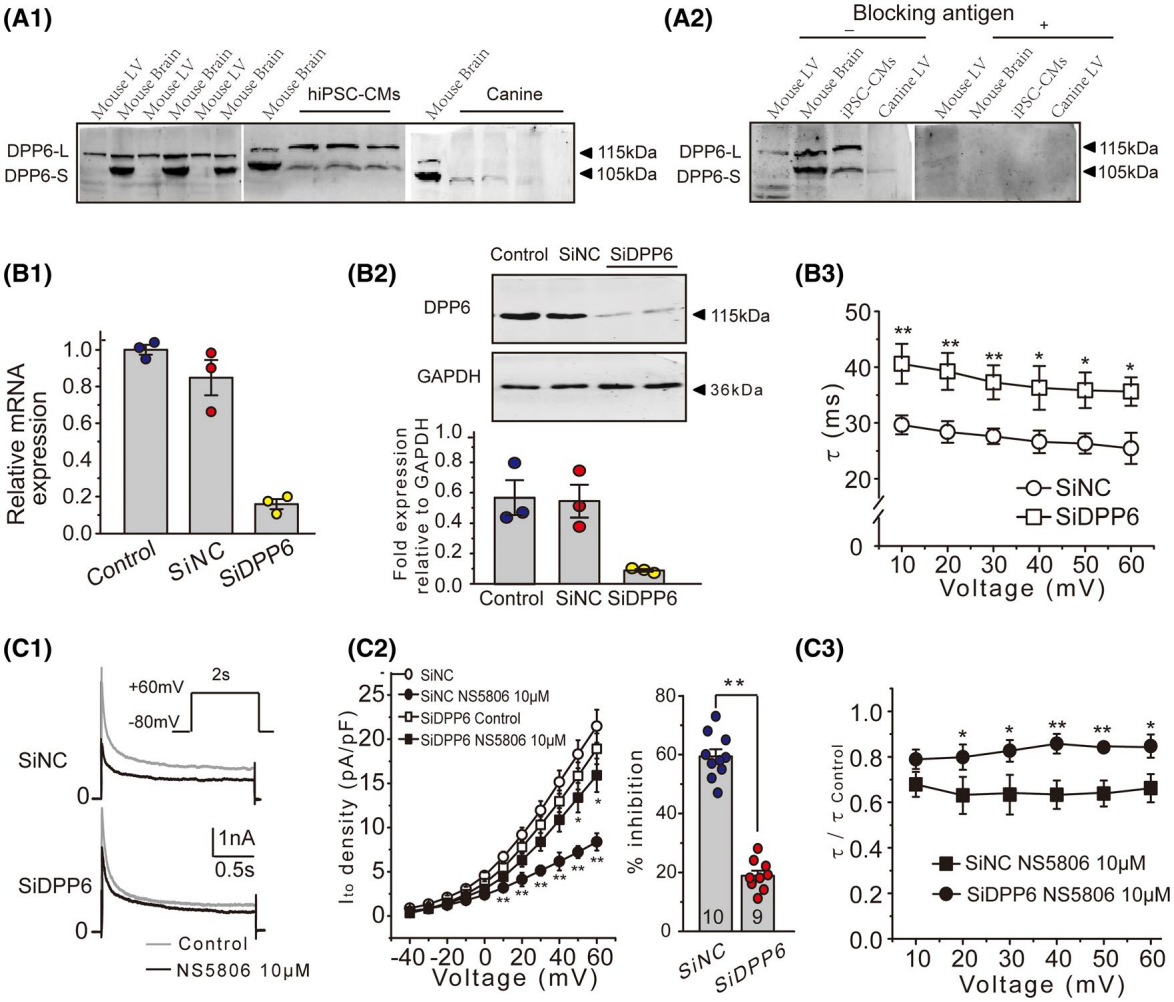
DPP6 expression in mouse, canine ventricular tissue, and hiPSC‐CMs. A_1_, Representative immunoblots of DPP6 protein expression in mouse left ventricle, canine left ventricle, and hiPSC‐CMs. A_2_, Test for antibody specificity by preincubation with the antigen peptide. B_1_, Efficiency of siRNA‐mediated DPP6 knockdown (SiDPP6) in hiPSC‐CMs quantified by RT‐PCR and compared to non‐targeting control siRNA (SiNC) (N = 3). B_2_, Representative immunoblots of DPP6 in hiPSC‐CMs treated with siRNA targeting DPP6 (N = 3). B_3_, Time constants (*τ*) of the current inactivation in hiPSC‐CMs treated with siRNA targeting DPP6 and a non‐targeting control. Data obtained by fitting a single exponential equation (n = 9, *P < .05, **P < .01 vs SiNC). C_1_, Representative *I*_to_ current traces recorded from the hiPSC‐CMs treated with SiNC or SiDPP6. C_2_, Effect of siRNA knockdown of DPP6 on the I–V relationships of *I*_to_ and on the efficacy of NS5806‐mediated current inhibition in hiPSC‐CMs (left). Summary of the inhibitory effect of 10 μM NS5806 on *I*_to_ in SiNC‐ or SiDPP6‐treated hiPSC‐CMs is shown on the right (n = 10, **P < .01). C_3_, Effect of DPP6 knockdown on the kinetics of *I*_to_ inactivation in the presence of 10 μM NS5806 in hiPSC‐CMs (*P < .05, **P < .01 vs SiNC)

To gain better understanding of the role of DPP6 in the pharmacological responses of Kv4 channels, we determined the protein abundance of DPP6 in ventricular myocardium of dog and mouse, as well as in human hiPSC‐CMs. An antibody that binds with both DPP6‐L and DPP6‐S was used. The brain tissue was used as a positive control for the western blots since both DPP6‐L and DPP6‐S are present in the brain.^11^, ^27^ As shown in Figure 5A1, two protein bands were detected in the brain, the smaller molecule was more abundant than the larger one. This finding is consistent with higher abundance of DPP6‐S, as compared to DPP6‐L, in the brain.^29^ Thus, we assumed that two bands seen in our western blot experiments correspond to DPP6‐S and DPP6‐L, respectively. Intriguingly, a different expression pattern of DPP6 isoforms was found among the species tested. Thus, DPP6‐L was dominantly expressed in both mouse ventricular myocardium and hiPSC‐CMs, while it was almost undetectable in canine ventricular myocardium (Figure 5A1). In addition, DPP6‐S was virtually undetectable in canine heart preparation (Figure 5A1). Specificity of the DPP6 antibody was tested by western blot after pre‐incubation with the blocking antigen, which eliminated both bands in brain and heart preparations (Figure 5A2). Notably, the expression level of KChIP2 protein was comparable among these species (Figure S6). Based on the above results we hypothesized that the dominant expression of DPP6‐L might be responsible for the inhibitory effect of NS5806 on native *I*_to_ in mouse cardiomyocytes and hiPSC‐CMs.

To test this hypothesis, we knocked down DPP6 expression in hiPSC‐CMs using a small interference RNA approach (the siRNA we used recognized both DPP6‐L and DPP6‐S mRNAs but since DPP6‐L is the predominant isoform in hiPSC‐CMs, we assume that the main effect of the knockdown would be mediated by the downregulation of DPP6‐L). The validity of siRNA DPP6 (SiDPP6) was confirmed by RT‐PCR (Figure 5B1) and western blot (Figure 5B2), in which scramble siRNA (SiNC) was used as a control. After the knock down of DPP6 in hiPSC‐CMs, the *I*_to_ displayed significantly slower inactivation kinetics, further evidencing a successful knockdown of DPP6 expression (Figure 5B3). Knockdown of DPP6 significantly reduced the response to NS5806, albeit the compound still produced a modest suppression of the current in hiPSC‐CMs (Figure 5C1,C2). Similarly, NS5806‐induced acceleration of the current inactivation was antagonized by DPP6 knockdown (Figure 5C3). These data provide further evidence that DPP6 subunits play a key role in the inhibitory action of NS5806 on native *I*_to_.

### 3.5 Analysis on the association between KChIP2 and DPP6

Although our findings revealed the key role of DPP6 subunits in defining pharmacological profile of *I*_to_, the fact that NS5806 failed to affect the current amplitude and inactivation kinetics in Kv4 and Kv4/DPP6 channels suggests that KChIP2 is also required for the modulatory effects of NS5806. Specifically, both DPP6 and KChIP2 are required for NS5806‐mediated channel inhibition, while KChIP2 alone confers channel potentiation by NS5806. We further hypothesized that the inhibitory effect of NS5806 on the Kv4 channel may depend on the association between DPP6‐L and KChIP2.

**Figure 6:**
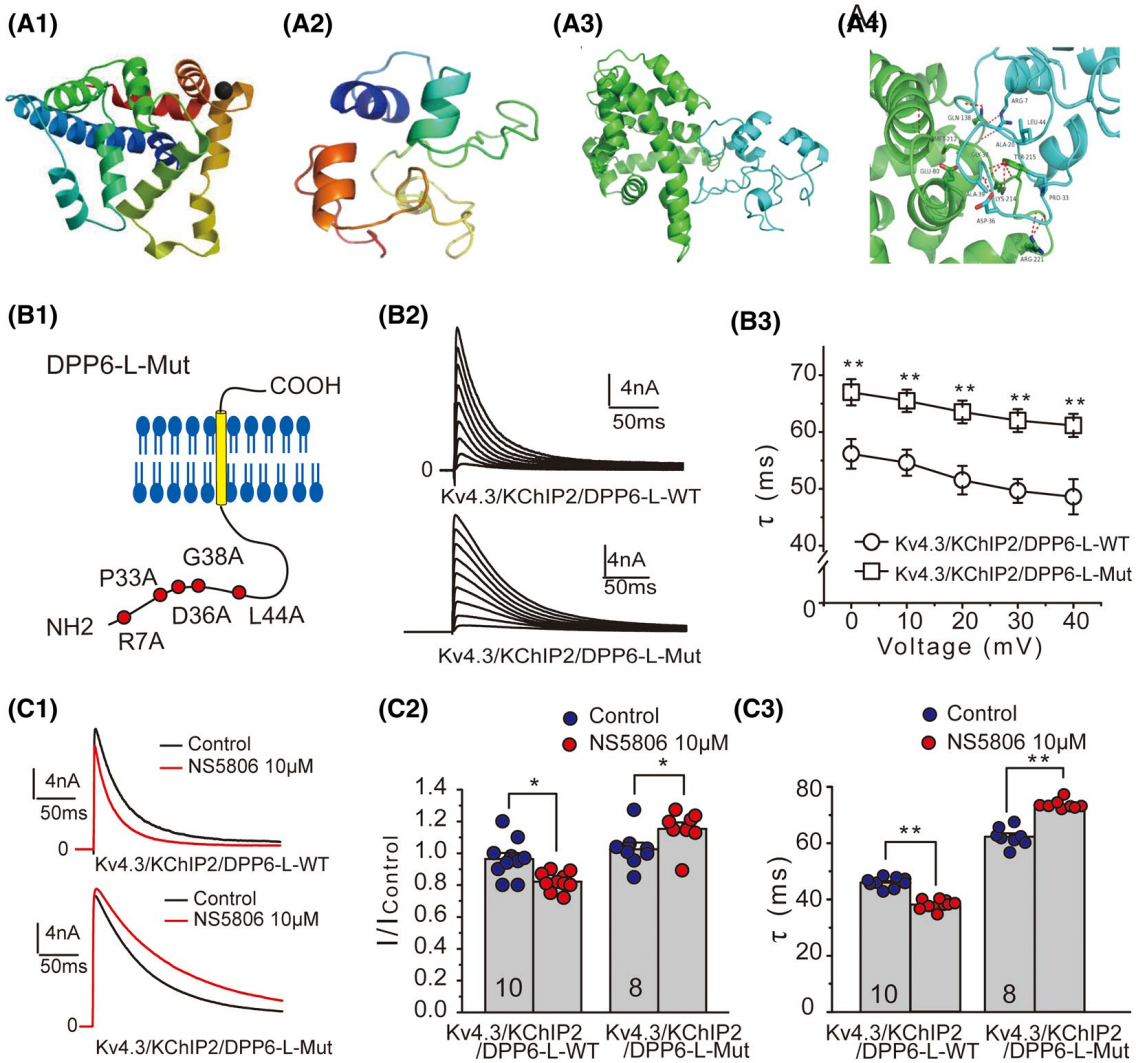
Analysis on the putative interactions between KChIP2 and DPP6‐L. A_1_, Modeling and docking simulation of putative interactions between DPP6‐Lin and KChIP2*.* Homology model of KChIP2. A_2_, The top‐ranked model of the intracellular domain of DPP6‐L (DPP6‐Lin). A_3_, Top‐ranked models of both proteins. A_4_, Best scored model of docking KChIP2 with DPP6‐Lin; putative‐interacting residues are indicated. B_1_, Schematic depiction of DPP6‐L and the location of mutated residues within the putative KChIP2 interaction site. B_2_, Representative recordings of Kv4.3/KChIP2/DPP6‐L‐WT and Kv4.3/KChIP2/DPP6‐L‐Mut currents from HEK293 cells using 500 ms square voltage pulses (from −40 to +40 mV; holding potential is −80 mV). B_3_, The time constants of inactivation (*τ*) of Kv4.3/KChIP2/DPP6‐L‐WT and Kv4.3/KChIP2/DPP6‐L‐Mut current traces plotted against voltage (**P < .01). C_1_, Representative recordings of *I*_Kv4.3/KChIP2/DPP6‐L‐WT_ and *I*_Kv4.3/KChIP2/DPP6‐L‐Mut_ in HEK293 cells before and after 10 μM NS5806. C_2_, Summary data for the effect of NS5806 on the current amplitudes of Kv4.3/KChIP2/DPP6‐L‐WT and Kv4.3/KChIP2/DPP6‐L‐Mut channels (*P < .05). C_3_, Summary data for the effect of NS5806 on the current inactivation kinetics (at +40 mV) of Kv4.3/KChIP2/DPP6‐L‐WT and Kv4.3/KChIP2/DPP6‐L‐Mut channels (**P < .01)

To identify the possible sites involved in association between two proteins, the structural models of KChIP2 and DPP6‐L were generated. Based on a known structure of KChIP4a,^23^ a homology model of KChIP2 was generated (Figure 6A1). The structure of intracellular domain of DPP6‐L (DPP6‐Lin) was predicted using *ab initio* protein prediction approach (Figure 6A2). Top‐ranked models of both proteins are shown in Figure 6A3. Then, molecular docking simulations were performed to identify possible binding sites between these two proteins. Ten models were generated in total and the model with the most favorable binding energy (Figure 6A4) was used as a template for site‐directed mutagenesis study. The detailed predicted results of interactions between DPP6‐Lin and KChIP2 in the model are shown in Table S4. Residues R7, P33, D36, G38, and L44 within the putative KChIP2‐associating site of DPP6‐L (Figure 6B1) and were mutated to alanines with an aim to generate a DPP6‐L mutant with reduced KChIP2‐binding affinity (DPP6‐L‐Mut). The current for Kv4.3/KChIP2/DPP6‐L‐WT and Kv4.3/KChIP2/DPP6‐L‐Mut (1:1:1) channels were then recorded and compared (Figure 6B2). The current inactivation kinetics of Kv4.3/KChIP2/DPP6‐L‐Mut was significantly slower, as compared to Kv4.3/KChIP2/DPP6‐L‐WT (Figure 6B3). The representative current traces of Kv4.3/KChIP2/DPP6‐L‐WT channels and Kv4.3/KChIP2/DPP6‐L‐Mut channels in the absence and presence of NS5806 are shown in Figure 6C1. Strikingly, the mutations conferred a reversal of the effect of NS5806 on the current amplitude: from inhibition of *I*_Kv4.2/KChIP2/DPP6‐L‐WT_ to modest (but significant) enhancement of *I*_Kv4.2/KChIP2/DPP6‐L‐Mut_ (Figure 6C2). In addition, mutations within the putative KChIP2 interaction sites also reversed the effect of NS5806 on the current inactivation kinetics (Figure 6C3). These experiments add further support to the hypothesis that an association between DPP6 and KChIP2 confers the inhibitory effect of NS5806 on Kv4 channel.

## 4 DISCUSSION

### 4.1 NS5806 suppressed Kv4‐generated *I*_to_ in mouse cardiomyocytes and hiPSC‐CMs

NS5806 has been put forward as a prototypic activator of *I*_to_.^12^, ^13^ Yet, the efficacy of the *I*_to_ enhancement by NS5806 varies significantly between the tissue types and even within different regions across the ventricular wall (ie, between epicardial, midmyocardial, or endocardial cells^15^, ^30^). The potentiating effects have also been demonstrated on heterologously expressed Kv4.3/Kv4.2 channels in the presence of KChIP2 with or without DPP6. In addition, NS5806 was shown to strongly inhibit cloned Kv1 family channels (Kv1.4/Kv1.5).^12^ A recent study has revealed that NS5806 markedly increased *I*_to_ amplitude in rabbit ventricular myocytes, but it inhibited *I*_to_ in rabbit atrial cells.^31^ These different responses are difficult to explain solely by contribution of Kv1.4 since Kv4.3, 4.2, and 1.4 are all expressed across the rabbit atria.^31^

Consistent with the previous finding, we observed a significant increase in *I*_to_ amplitude in the presence of NS5806 in canine ventricular myocytes. However, the effect of NS5806 on native *I*_to_ in mouse ventricular cardiomyocytes as well as in hiPSC‐CMs was strikingly different as current inhibition was seen instead. In mouse ventricles, Kv4.2/Kv4.3 subunits conduct *I*_to,f_, whereas Kv1.4 forms *I*_to,s_ channels.^32^ Using specific Kv4 channel blocker combined with kinetic differentiation, our experiments further confirmed that *I*_to,f_ was specifically inhibited by NS5806 in mouse ventricular myocytes and hiPSC‐CMs. In both these cell types, NS5806 significantly accelerated current inactivation and shifted voltage dependence of steady‐state inactivation to more negative potentials. These effects on channel gating could account for the decrease in current amplitude.

Consistent with these results, a previous report has shown that NS5806 at 10 μM fails to enhance, but rather produced a small reduction of *I*_to_ amplitude in hiPSC‐CMs.^17^ Our western blot analysis provided further evidence for a dominant expression of Kv4 in hiPSC‐CMs, while only a trace amount of Kv1.4 was found in these cells. This observation is seemingly at odds with the previous study^17^ showing presence of Kv1.4 mRNA in hiPSC‐CM; however, the abundance of Kv1.4 protein in hiPSC‐CM was not investigated in that study. The different expression pattern of ion channels may depend on different maturation state of hiPSC‐CMs. Yet, variable presence of Kv1.4 brings further complexity to *I*_to_ modulation by NS5806 since an inhibitory effect of NS5806 on cloned Kv1.4 channel has been demonstrated.^12^ Nevertheless, the fact that there were comparable levels of Kv1.4 protein abundance in mouse and canine ventricular tissue (while the effects of NS5806 on *I*_to_ were opposite) suggests that the effect of NS5806 on the Kv4 channel complex is a dominant mechanism of the *I*_to_ modulation in the heart.

### 4.2 DPP6 and KChIP2 subunits confer the modality of *I*_to_ channel response to NS5806

We think that involvement of different auxiliary subunits could offer an explanation for the difference in NS5806 action in cardiac cells from different species. The KChIP2 and DPP6 are most likely candidates for auxiliary subunits of Kv4‐containing *I*_to_ channel complex.^9^ We thus tested the effect of NS5806 on cloned Kv4 with KChIP2 and DPP6 subunits in HEK293 cells. In agreement with the previous reports,^12^ our results indicate that NS5806 significantly enhanced the current amplitude of Kv4.3/KChIP2 and Kv4.3/KChIP2/DPP6‐S channels, and concomitantly slowed the inactivation kinetics of these channel complexes. On the contrary, NS5806 markedly decreased the peak amplitudes of currents mediated by Kv4.3/KChIP2/DPP6‐L or Kv4.2/KChIP2/DPP6‐L channel complexes and significantly sped the inactivation of these channels. The above effect of NS5806 was further potentiated when DPP6‐L expression level was elevated. These changes recapitulated the effect of NS5806 on native *I*_to_ in the mouse ventricular cells. The results suggest that auxiliary subunits DPP6‐L and KChIP2 confer the opposite responses to NS5806. Yet, KChIP2 was also necessary for the inhibitory action of NS5806 on *I*_to_ as Kv4/DPP6‐L complex was insensitive to the compound (Figure 4A,B).

Consistent with the findings from heterologous expression system, our western blot analysis demonstrated that DPP6‐L was dominantly expressed in both mouse ventricular myocardium and iPSC‐CMs. In contrast, in canine ventricular myocardium, DPP6‐L presence was undetectable while low levels of DPP6‐S expression were seen. Levels of KChIP2 expression were comparable between all three species (Figure 5A). Furthermore, siRNA knockdown of DPP6 significantly antagonized the NS5806‐induced reduction of current amplitude and acceleration of inactivation of native *I*_to_ in hiPSC‐CMs. Thus, our data clearly demonstrate that the inhibitory response to NS5806 in cardiomyocytes depends on the DPP6‐L subunit. The effects of NS5806 on cloned Kv4 channel complexes and native *I*_to_ reported here and in previous reports are summarized in Table 1.

**Table 1:**
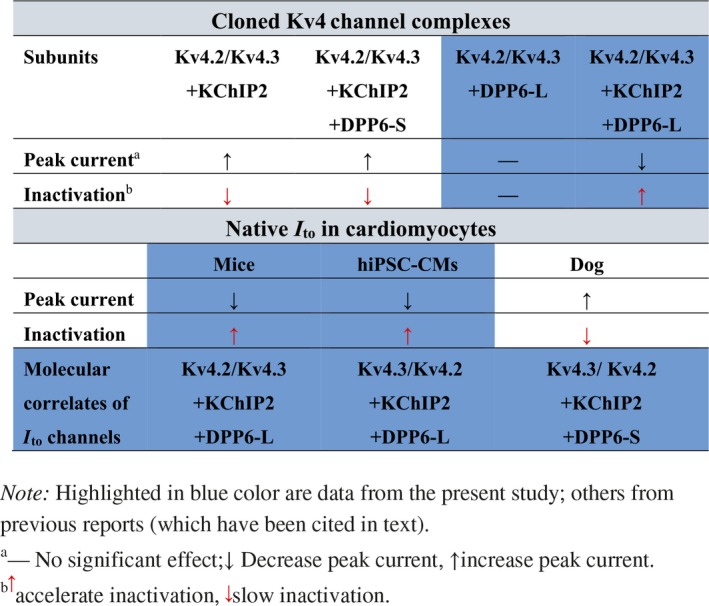
Summary for the effects of NS5806 on cloned Kv4 channel complexes and native *I*_to_ channels

### 4.3 Possible molecular mechanism underlying the inhibitory response to NS5806

Previous study suggested that the potentiating effect of NS5806 on Kv4 channels depends on the accessory protein KChIP2.^12^ KChIP2 is a cytosolic protein that interacts with the intracellular N‐termini of Kv4 subunits^33^ and results in a slower inactivation kinetics and acceleration of recovery from inactivation.^10^ A recent study has shown that NS5806 binds to hydrophobic residues on the C‐terminus of KChIP3 and increases the affinity between KChIP3 and the N‐terminus of Kv4.3.^13^ It is likely that the similar mechanism underlies the effect of NS5806 on Kv4/KChIP2 complex channel since KChIP2 and KChIP3 have nearly identical NS5806‐binding sites. However, how does DPP6‐L confer the inhibitory effect of NS5806 on Kv4/KChIP2/DPP6‐L channel complex? The DPP6 is a single transmembrane subunit consisting of a short intracellular N‐terminal domain and a long extracellular C‐terminal domain.^8^ When co‐expressed with Kv4 subunits, DPP6 (either long or short isoform) increases the rate of inactivation of a resulting channel complex, negatively shifts the voltage dependence of steady‐state inactivation, and increases the rate of recovery from inactivation.^11^ Interestingly, the present study revealed that NS5806 produces biophysically very similar effects on both native *I*_to_ channels in tissues expressing DPP6‐L (murine and human but not canine) and on recombinant Kv4/KChIP2/DPP6‐L channel complexes. It is thought that the discrete and specific interactions mediate the effects of KChIP and DPP subunits on the gating of Kv4 channels.^34^ Thus, we speculate that the inhibitory effect of NS5806 on the *I*_to_ current might result from the increased binding affinity between DPP6‐L and the Kv4/KChIP2 complex. One explanation for the observed effects might be in that (a) intracellular N‐terminal domain of DPP6‐L interacts with KChIP2, (b) the association between two subunits exposes NS5806‐binding site in DPP6‐L while hindering the binding of NS5806 to KChIP2 subunits. In support of this scenario, the molecular docking simulations indicated that several putative residues, including R7, P33, D36, G38, and L44 in the N‐terminus of DPP6‐L, are likely to associate with the C‐terminus of KChIP2. Mutation of these residues reversed the inhibitory effect of NS5806 into potentiation of the *I*_to_ current. In addition, the acceleration of inactivation was turned into slowing down. Interestingly, according to the docking simulation, the putative KChIP2‐DPP6‐L association site is predominantly localized in the C‐terminus of KChIP2, which has been reported to contain also the binding site for NS5806.^13^ This may be the reason why the association between two subunits could hamper binding of NS5806 to KChIP2 and promote binding to DPP6‐L instead. Binding of NS5806 to DPP6‐L might facilitate its association with the pore‐forming Kv4 subunit and “switch” the potentiation of *I*_to_ into inhibition. Interestingly, previous studies reported that the peak amplitudes of currents mediated by ternary Kv4/KChIP2/DPP6‐S channel complexes were potentiated and the current inactivation significantly slowed by NS5806.^12^, ^28^ DPP6–S isoform lacks 62 N‐terminal residues that form putative KChIP‐interacting site. Hence, the ability of DPP6‐S to associate with KChIP2 is likely to be compromised. This is a likely explanation for the divergent results observed with the DPP6‐S and DPP6‐L isoforms. Indeed, our data predict that similarly to the DPP6‐L‐Mut (Figure 6), the DPP6‐S isoform would not be able to bind to KChIP2 and, thus, to switch the modality of NS5806 response of the *I*_to_ channel complex. However, further structural insights are needed to confirm the direct interaction between KChIP2 and DPP6‐L. In addition, KCNE β‐subunits also regulate Kv4 channels in the hearts of some species and may influence the response to channel modulator.^35^ Further studies are needed to test the possibility of the involvement of KCNE subunits in the NS5806 response.

## 5 CONCLUSION

This study discovers molecular mechanism for the opposing response of cardiac Kv4 channel complex to its modulator NS5806. We show that the alternative assembly of the complex with different auxiliary subunits in different species results in channels with strikingly different biophysical and pharmacological properties. These findings provide novel insight for the development of new ion channel modulators for treatment of cardiovascular diseases.

## CONFLICT OF INTEREST

The authors report no conflict of interest.

## AUTHOR CONTRIBUTIONS

Hongxue Zhang, Hua Zhang, C. Wang, Y. Wang, R. Zou, C. Shi, and B. Guan performed the research. Y. Xu and N. Gamper designed the research strategy. Hongxue Zhang, C. Wang, R. Zou, and C. Shi analyzed the data. Hongxue Zhang, N. Gamper, and Y. Xu wrote the paper.
