# Supplementary material for: Auxiliary subunits control biophysical properties and response to compound NS5806 of the Kv4 potassium channel complex
Source: FASEB J. 2019 Nov 27;34(1):807–21. doi: 10.1096/fj.201902010RR (PMC6972550; doi:10.1096/fj.201902010RR)
Supplement: Supplementary file 1 [file FSB2-34-807-s001.doc]

**Supplementary Materials**

**Auxiliary subunits control biophysical properties and response to compound NS5806 of the Kv4 potassium channel complex**

Running title: β-subunits control pharmacology of Kv4 potassium channel

Hongxue Zhang^a^, Hua Zhang^a^, Chanjuan Wang^a^, Yuhong Wang^b^, Ruya Zou^a^, Chenxia Shi^a^, Bingcai Guan^a^, Nikita Gamper^c,a^*, Yanfang Xu^a^*

^a^Department of Pharmacology, Hebei Medical University; The Key Laboratory of New Drug Pharmacology and Toxicology, Hebei Province; The Key Laboratory of Neural and Vascular Biology, Ministry of Education, Shijiazhuang 050017, China

^b^Institute of Masteria Medica, Chinese Academy of Medical Sciences&Peking Union Medical College, Beijing 100050, China

^c^Faculty of Biological Sciences, University of Leeds, Leeds, UK

*Correspondence should be sent to

Yanfang Xu

Department of Pharmacology,

Hebei Medical University, Hebei, China

Tel.: 86-311-86266431

E-mail: yanfangxu@hebmu.edu.cn

Nikita Gamper

School of Biomedical Sciences

Faculty of Biological Sciences

University of Leeds

Leeds, UK

Tel: +44 (0) 113 343 7923

Email: [n.gamper@leeds.ac.uk](mailto:n.gamper@leeds.ac.uk)

**Supplemental Figures**

**
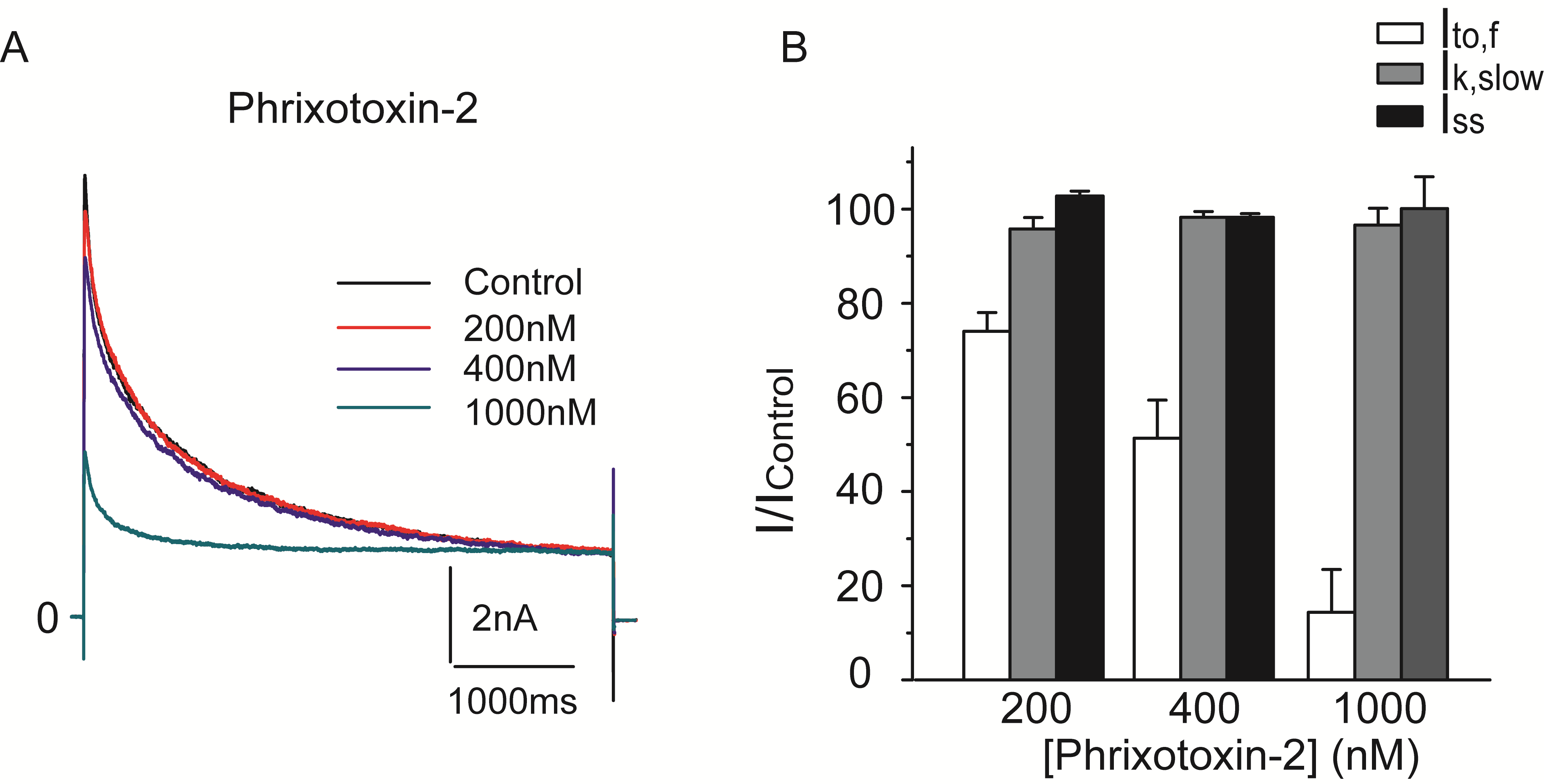
**

**Fig.S1. Effects of Phrixotoxin-2 on outward potassium current in mouse left ventricular myocytes.** (A) Representative traces of outward potassium currents after the bath application of Phrixotoxin-2 at different concentrations (as indicated). (B) Concentration-dependent effect of Phrixotoxin-2 on the three components of K^+^ current: *I*_to,f_, *I*_k,slow_ and *I*_ss_, differentiated as described in Methods (n=20, N=5).

**
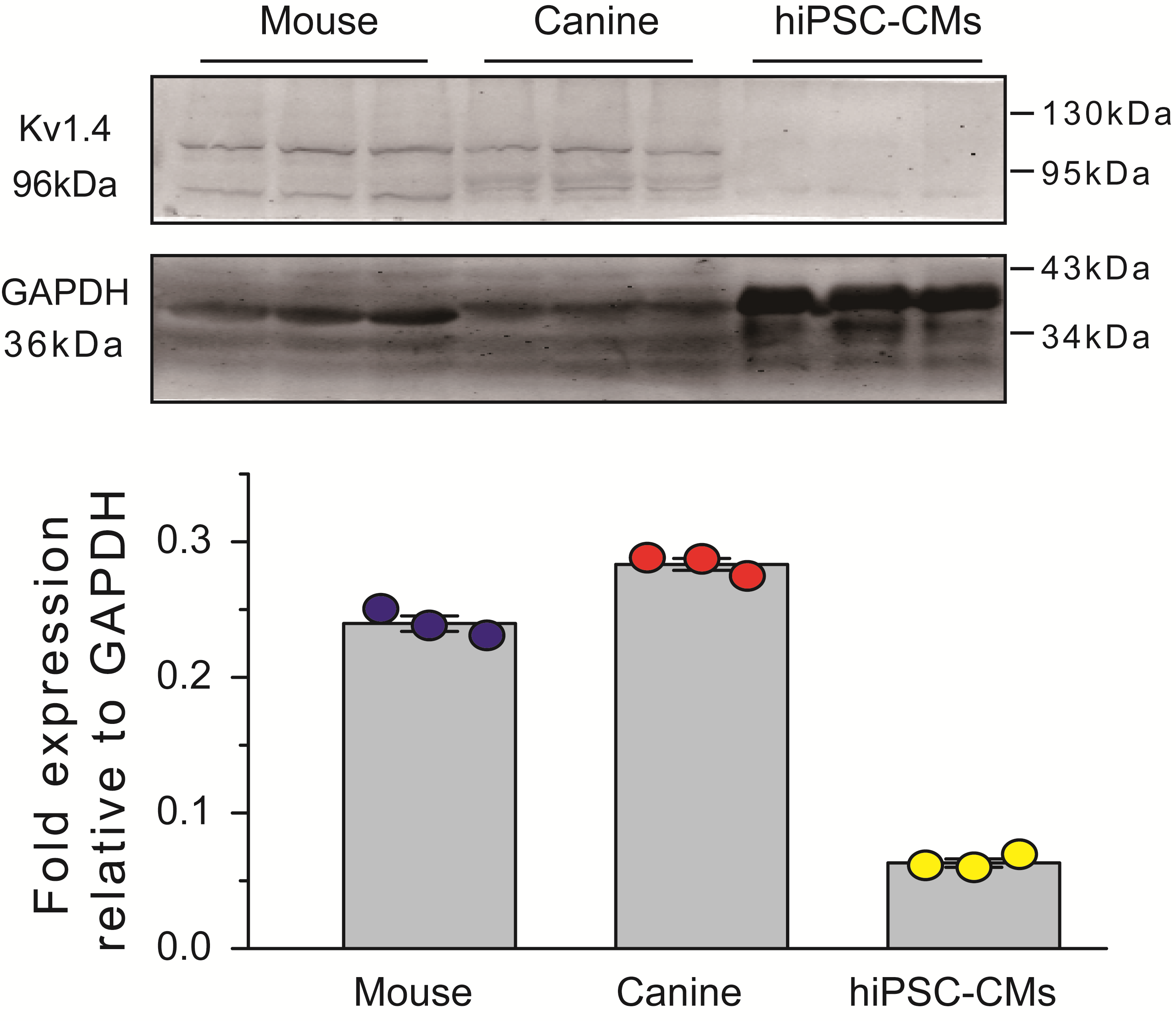
**

**Fig.S2 Western-blot analysis of Kv1.4 in mouse and canine ventricular tissue and in hiPSC-CMs.** Upper panel displays representative western blot bands from three type of tissue/cells (each type with 3 samples). Bottom panel depicts summary of densitometric quantification, normalized to GAPDH (N=3).


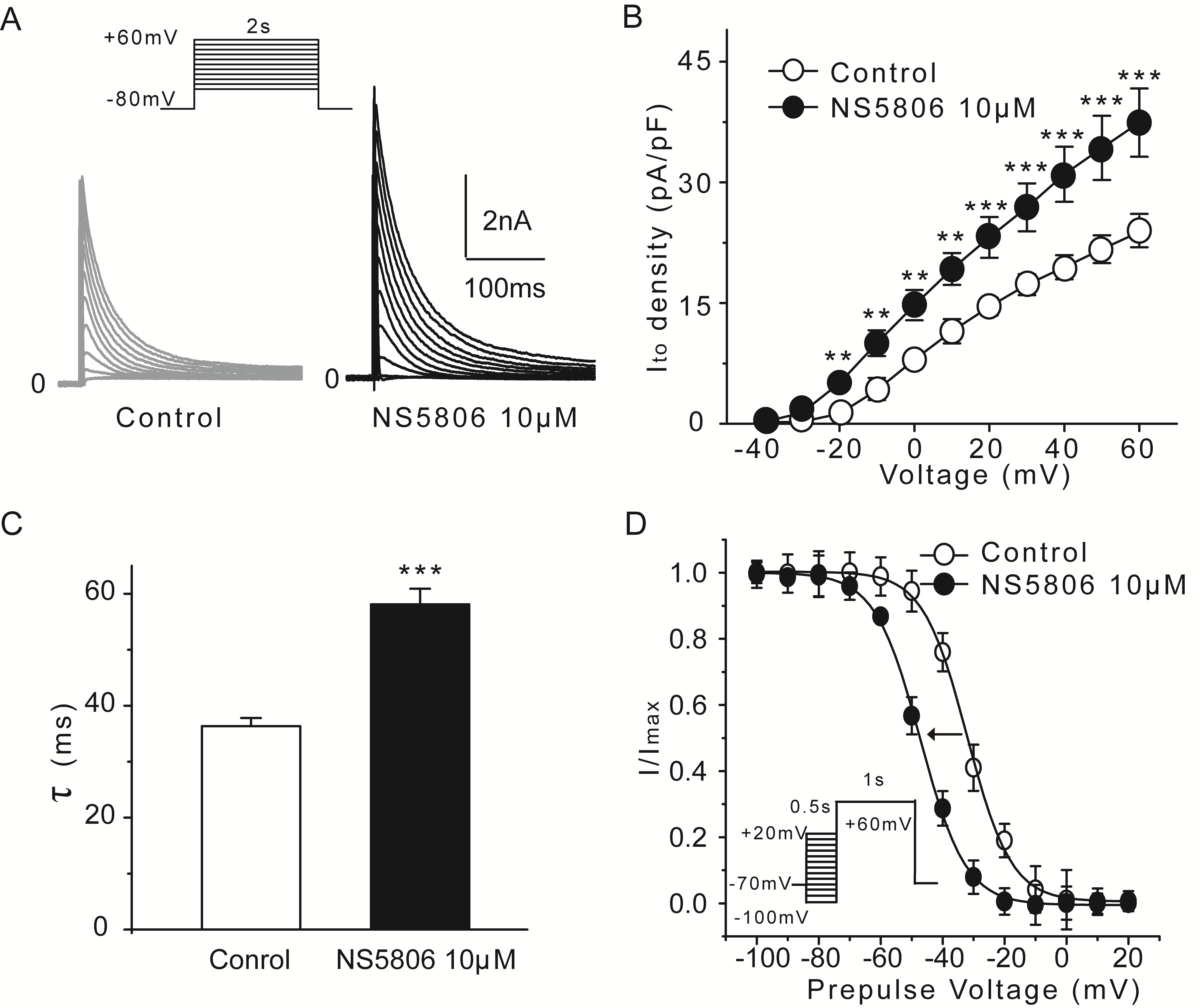


**Fig. S3. Effects of NS5806 on I_to_ current in canine left ventricular myocytes.** (A) Representative *I*_to_ current traces recorded in the absence and presence of 10 μM NS5806 using the voltage protocol shown in the inset. (B) Mean I-V relations of the *I*_to_ current (expressed as current density) before and after 10 μM NS5806 (n=12, N=3, ****P*<0.001 *vs.* Control). (C) Mean time constant of inactivaion (τ) in absence and presence of 10 μM NS5806 at +60 mV. (n=12, N=3, ****P*<0.001 *vs.* Control). (D) Effect of NS5806 on the voltage dependence of *I*_to_ inactivation; normalized peak current (Mean ± SEM) is plotted against the conditioning prepulse voltage (voltage protocol is shown in the inset) in control conditions and in the presence of 10μM NS5806. Data were fitted by the Boltzmann function.


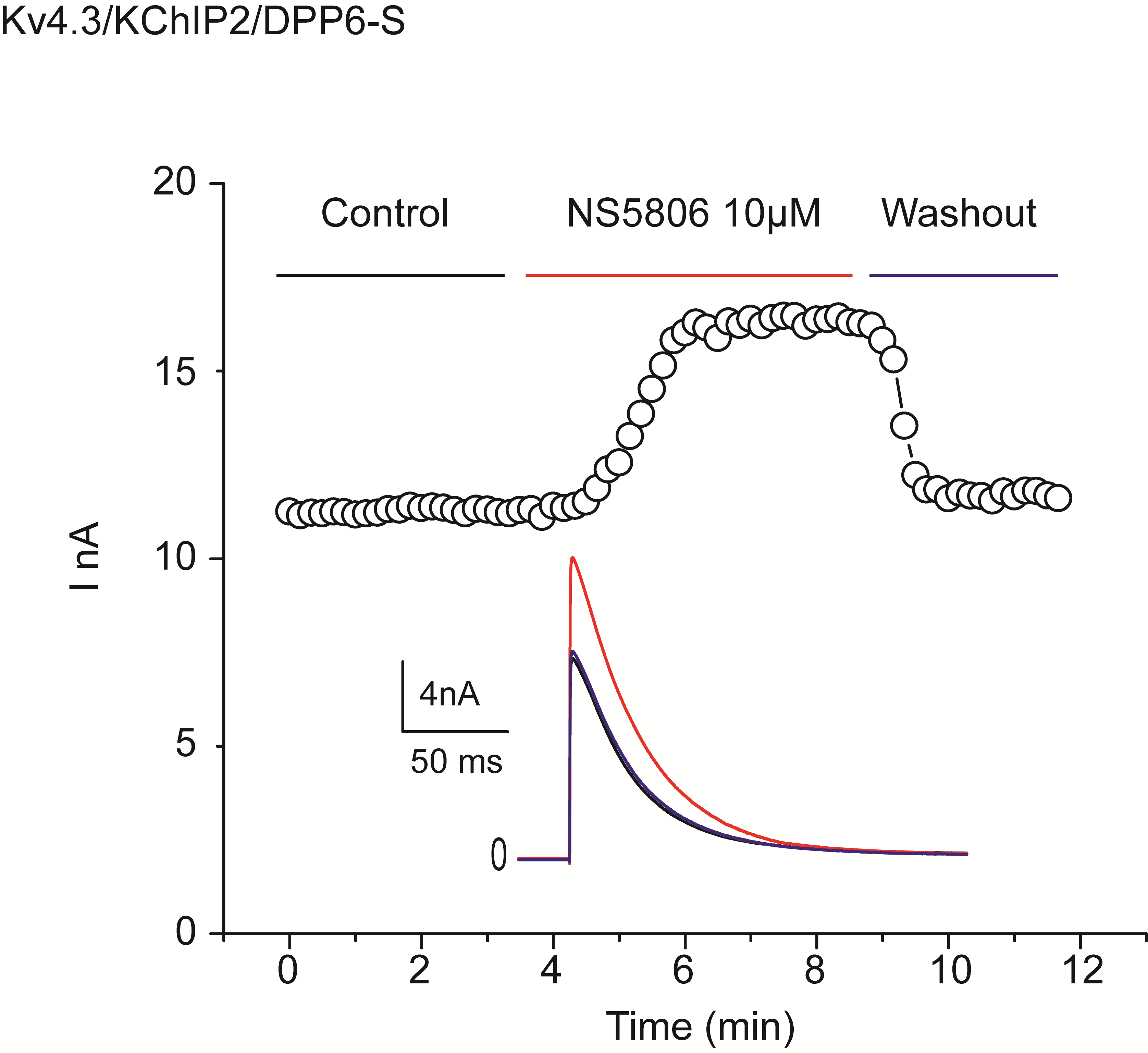


**Fig. S4. Effect of NS5806 on cloned Kv4.3/KChIP2/DPP6-S channels in HEK293 cells.** A short variant of DPP6 was used with the transfection ratios of Kv4.3/KChIP2/DPP6-S subunit 1:1:1.


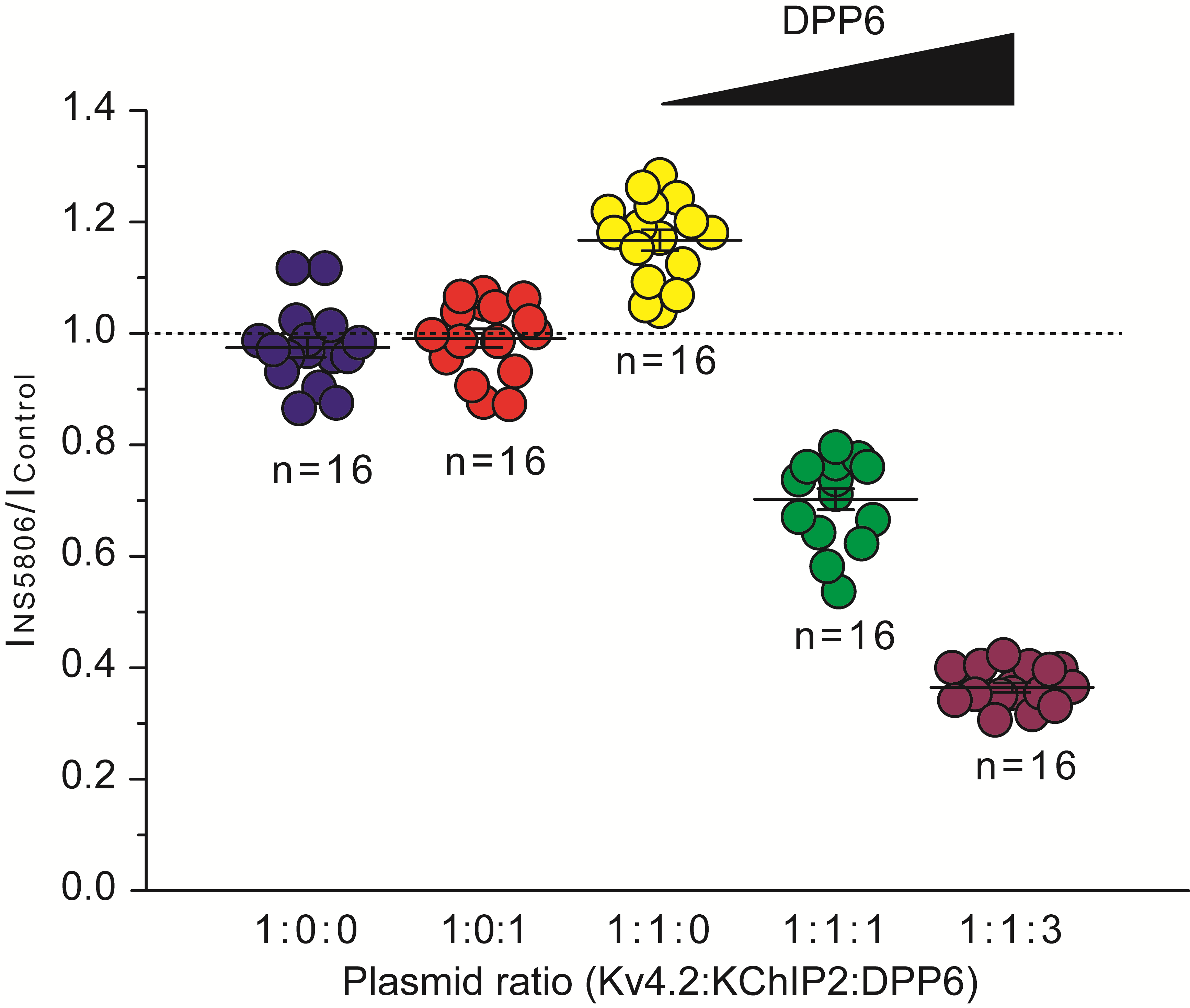


**Fig. S5. Effect of NS5806 on cloned Kv4.2/KChIP2/DPP6-L channels in HEK293 cells.** Shown are the effects of 10 μM NS5806 on the Kv4.2/KChIP2/DPP6-L currents produced by different subunit transfection ratios, measured at +40 mV.


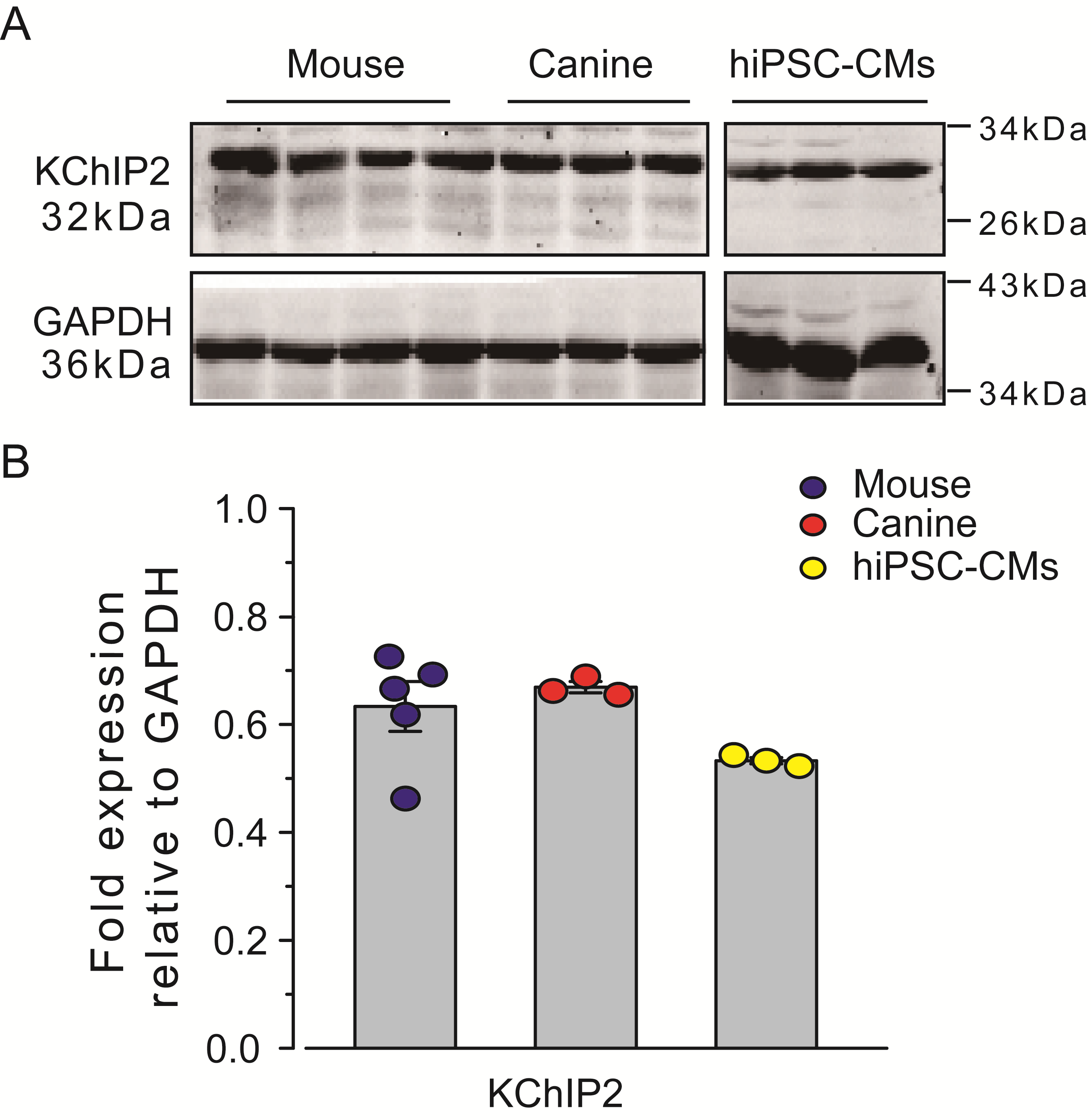


**Fig. S6. KChIP2 expression in mouse, canine ventricular tissue and hiPSC-CMs.** A. Representative immunoblots of KChIP2 protein expression. B. Summary data for KChIP2 densitometry quantification normalized to GAPDH.

**Supplementary Tables**

**Table S1.**  **Effect of 10 μM NS5806 on the peak currents of Kv4.3/KChIP2/DPP6-L channels with different transfection plasmid ratio**

| Peak current (nA)  Voltage  (mV) | 1:0:0 (n=16) | | 1:0:1 (n=16) | | 1:1:0 (n=22) | | 1:1:1(n= 22) | | 1:1:3(n=22 ) | |
| --- | --- | --- | --- | --- | --- | --- | --- | --- | --- | --- |
|  | Control | NS5806 | Control | NS5806 | Control | NS5806 | Control | NS5806 | Control | NS5806 |
| 0 | 3.1±0.4 | 3.1±0.9 | 3.3±1.0 | 4.1±1.3 | 4.0±1.6 | 6.3±0.9* | 7.8±0.5 | 6.2±0.3* | 7.0±1.1 | 3.1±0.6* |
| 10 | 4.7±0.4 | 4.9±1.1 | 5.7±0.6 | 6.1±1.7 | 6.6±0.8 | 9.3±0.9* | 9.8±0.7 | 7.8±0.4* | 9.1±1.0 | 3.6±0.6** |
| 20 | 6.7±1.6 | 6.8±1.5 | 8.2±0.7 | 8.5±0.8 | 9.4±0.9 | 12.4±1.1* | 11.8±0.8 | 9.5±0.4* | 11.0±1.2 | 4.3±0.7** |
| 30 | 8.8±1.8 | 8.6±2.3 | 10.5±1.2 | 11.1±0.8 | 12.3±1.1 | 15.7±1.6* | 13.9±0.9 | 11.2±0.5** | 12.8±1.4 | 5.1±0.8** |
| 40 | 10.4±2.3 | 10.7±1.1 | 13.1±2.4 | 13.8±1.5 | 15.5±0.5 | 18.9±0.9** | 16.1±1.1 | 12.8±0.6** | 14.8±1.5 | 5.8±0.9*** |

^*^*P*<0.05, ^**^*P* <0.01, ^***^*P*<0.001 vs Control.

**Table S2. Effect of 10 μM NS5806 on the inactivation kinetics of Kv4.3/KChIP2/DPP6-L** channels with different transfection plasmid ratio

| Decay τ (ms)  Voltage  (mV) | 1:0:0 (n=22) | | 1:0:1(n=22) | | 1:1:0 (n=22) | | 1:1:1(n=22) | | 1:1:3(n=22) | |
| --- | --- | --- | --- | --- | --- | --- | --- | --- | --- | --- |
|  | Control | NS5806 | Control | NS5806 | Control | NS5806 | Control | NS5806 | Control | NS5806 |
| 0 | 46.2±0.8 | 46.8±1.5 | 38.1±0.8 | 36.1±0.3 | 66.4±2.3 | 75.1±1.4* | 55.1±1.7 | 43.3±1.5* | 35.4±2.1 | 20.1±1.1* |
| 10 | 45.8±1.4 | 46.9±1.1 | 37.8±0.6 | 35.2±0.6 | 64.6±1.3 | 75.9±1.1** | 53.6±0.9 | 39.2±1.3** | 34.7±0.5 | 20.2±0.5** |
| 20 | 46.1±1.3 | 47.3±1.4 | 36.5±0.3 | 35.9±0.4 | 63.5±0.9 | 73.2±0.7** | 50.5±1.3 | 36.7±2.1** | 33.3±1.4 | 19.5±0.7** |
| 30 | 46.6±0.4 | 47.6±1.4 | 36.3±0.8 | 33.0±0.7 | 62.0±1.4 | 72.3±1.2** | 48.6±0.7 | 38.6±1.2** | 32.3±0.6 | 18.6±0.2** |
| 40 | 46.3±0.8 | 48.2±0.6 | 34.2±0.3 | 33.1±0.4 | 63.3±1.0 | 71.7±0.4** | 47.1±0.4 | 37.6±0.4** | 34.2±0.3 | 19.7±0.2** |

^*^*P*<0.05, ^**^*P* <0.01 vs Control.

**Table S3. Effect of 10 μM NS5806 on the peak currents of Kv4.2/KChIP2/DPP6-L** channels with different transfection plasmid ratio

| Peak current (nA)  Voltage  (mV) | 1:0:0 (n=16) | | 1:0:1 (n= 16) | | 1:1:0 (n=16) | | 1:1:1(n=16) | | 1:1:3 (n=16) | |
| --- | --- | --- | --- | --- | --- | --- | --- | --- | --- | --- |
|  | Control | NS5806 | Control | NS5806 | Control | NS5806 | Control | NS5806 | Control | NS5806 |
| 0 | 1.7±0.8 | 2.0±0.3 | 3.6±1.0 | 4.2±0.3 | 7.2±1.4 | 7.6±1.7 | 6.1±0.6 | 4.4±1.1* | 3.9±0.6 | 1.3±0.3** |
| 10 | 3.3±0.9 | 3.8±0.3 | 4.9±1.2 | 5.7±0.9 | 7.9±0.9 | 8.8±1.9* | 7.8±1.0 | 5.8±1.2* | 5.9±0.9 | 2.2±1.9** |
| 20 | 5.5±1.0 | 5.5±0.5 | 6.3±1.5 | 7.3±0.2 | 8.9±0.9 | 10.3±2.1* | 10.3±1.2 | 7.8±1.7* | 8.1±1.5 | 2.6±1.1** |
| 30 | 7.6±1.1 | 7.4±1.8 | 8.1±2.1 | 9.0±1.5 | 10.3±1.4 | 12.5±1.8** | 12.7±1.1 | 9.8±2.1** | 10.4±1.0 | 3.5±1.6** |
| 40 | 9.8±1.3 | 9.4±2.7 | 10.5±1.1 | 11.3±1.4 | 12.2±0.8 | 15.3±2.3** | 15.4±2.6 | 11.7±2.6** | 13.5±1.1 | 4.7±1.8*** |

^*^*P*<0.05, ^**^*P* <0.01, ^***^*P*<0.001 vs Control.

**Table S4.** **Predicted** **results of interactions between DPP6-Lin and KChIP2 in a model with the most favored binding energy.**

| Pair* | Residue No. in DPP6in | Residue in DPP6in | Atom in DPP6in | Residue No. in KChIP2 | Residue in KChIP2 | Atom in KChIP2 | Distance |
| --- | --- | --- | --- | --- | --- | --- | --- |
| 1 | 7 | ARG | NE | 138 | GLN | OE1 | 2.92 |
| 2 | 7 | ARG | NH1 | 212 | MET | O | 3.53 |
| 3 | 7 | ARG | NH1 | 138 | GLN | OE1 | 2.88 |
| 4 | 38 | GLY | N | 215 | TYR | OH | 2.56 |
| 5 | 39 | ALA | N | 80 | GLU | OE2 | 3.87 |
| 6 | 44 | LEU | N | 215 | TYR | OH | 3.72 |
| 7 | 20 | ALA | O | 215 | TYR | N | 3.42 |
| 8 | 20 | ALA | O | 214 | LYS | N | 3.62 |
| 9 | 33 | PRO | O | 221 | ARG | NH1 | 3.46 |
| 10 | 36 | ASP | O | 215 | TYR | OH | 3.38 |

*Ten hydrogen bonds were predicted by the model; no disulfide bonds, covalent bonds, or salt bridges were found. Interface Area (Å^2^ ): 994.7. ∆G (kcal/mol): -8.9.
